# LncRNA SATB2-AS1 inhibits tumor metastasis and affects the tumor immune cell microenvironment in colorectal cancer by regulating SATB2

**DOI:** 10.1186/s12943-019-1063-6

**Published:** 2019-09-06

**Authors:** Mu Xu, Xueni Xu, Bei Pan, Xiaoxiang Chen, Kang Lin, Kaixuan Zeng, Xiangxiang Liu, Tao Xu, Li Sun, Jian Qin, Bangshun He, Yuqin Pan, Huiling Sun, Shukui Wang

**Affiliations:** 1 General Clinical Research Center, Nanjing First Hospital, Nanjing Medical University, No. 68, Changle Road, Nanjing, 210006 China; 2 School of Medicine, Southeast University, Nanjing, 210009 China; 3 Department of Laboratory Medicine, Nanjing First Hospital, Nanjing Medical University, Nanjing, 210006 China; 4 Department of Laboratory Medicine, The Second Affiliated Hospital of Nanjing Medical University, Nanjing, 210011 China

**Keywords:** SATB2-AS1, Colorectal cancer, SATB2, Metastasis, Immune response, WDR5, GADD45A

## Abstract

**Background:**

Emerging studies suggest that long non-coding RNAs (lncRNAs) play crucial roles in colorectal cancer (CRC). Here, we report a lncRNA, SATB2-AS1, which is specifically expressed in colorectal tissue and is significantly reduced in CRC. We systematically elucidated its functions and possible molecular mechanisms in CRC.

**Methods:**

LncRNA expression in CRC was analyzed by RNA-sequencing and RNA microarrays. The expression level of SATB2-AS1 in tissues was determined by quantitative reverse transcription polymerase chain reaction (qRT-PCR) and in situ hybridization (ISH). The functional role of SATB2-AS1 in CRC was investigated by a series of in vivo and in vitro assays. RNA pull-down, RNA immunoprecipitation (RIP), chromatin immunoprecipitation (ChIP), chromatin isolation by RNA purification (ChIRP), Bisulfite Sequencing PCR (BSP) and bioinformatics analysis were utilized to explore the potential mechanisms of SATB2-AS1.

**Results:**

SATB2-AS1 is specifically expressed in colorectal tissues and downregulated in CRC. Survival analysis indicates that decreased SATB2-AS1 expression is associated with poor survival. Functional experiments and bioinformatics analysis revealed that SATB2-AS1 inhibits CRC cell metastasis and regulates TH1-type chemokines expression and immune cell density in CRC. Mechanistically, SATB2-AS1 directly binds to WDR5 and GADD45A, *cis*-activating SATB2 (Special AT-rich binding protein 2) transcription via mediating histone H3 lysine 4 tri-methylation (H3K4me3) deposition and DNA demethylation of the promoter region of SATB2.

**Conclusions:**

This study reveals the functions of SATB2-AS1 in CRC tumorigenesis and progression, suggesting new biomarkers and therapeutic targets in CRC.

**Electronic supplementary material:**

The online version of this article (10.1186/s12943-019-1063-6) contains supplementary material, which is available to authorized users.

## Background

CRC is the third most common cancer by incidence and the fourth leading cause of cancer death worldwide [1]. Although some advances have been made in its diagnosis and treatment, patients with advanced CRC have a poor prognosis. Recurrence, metastasis and drug resistance are common causes of poor prognosis in patients with CRC [2]. Molecular and genetic alterations exert vital roles in these events and provide potential targets for therapy [3]. Therefore, a better understanding of the molecular mechanism underlying CRC progression is essential for the development of novel biomarkers and effective treatment methods for CRC.

LncRNAs are a class of transcripts with lengths greater than 200 nucleotides and no protein coding capacity [4]. Accumulating evidence reveals that lncRNAs are important regulators of gene expression and are involved in various physiological and pathological processes, including the occurrence and development of tumors [5]. The functions and underlying mechanisms of several lncRNAs in CRC have been reported [6, 7]. However, more specific roles of lncRNAs in CRC tumorigenesis and progression need to be explored.

In this study, we analyzed the genome-wide lncRNA expression profiles of CRC and adjacent normal tissue in two independent cohorts. We identified a novel lncRNA, SATB2-AS1, that was expressed at significantly lower levels in CRC and was associated with the prognosis of CRC. We assessed its biological functions using several in vitro and in vivo assays. We found that SATB2-AS1 could inhibit CRC metastasis and regulate TH1-type chemokines secretion in CRC cells. Mechanistic studies revealed that SATB2-AS1 might serve as a scaffold for WDR5 and GADD45A and recruit them to the promoter region of SATB2. This RNA-protein complex could activate SATB2 transcription by inducing DNA demethylation and histone H3K4me3 enrichment in the promoter region of SATB2. Taken together, this study unveils the clinical impact, biological roles and underlying mechanisms of SATB2-AS1 in CRC. SATB2-AS1 might be a promising biomarker and therapeutic target for CRC.

## Methods

### RNA sequencing and microarray data analysis

CRC gene expression data were obtained from the TCGA and GEO database. The independent datasets from GSE9348 (Hong Colorectal) [8], GSE8671 (Marra Colorectal) [9], GSE39582 (Marisa Colorectal) [10], GSE17538 (Smith Colorectal) [11], GSE14333 (Sieber Colorectal) [12], GSE2109 (EXPO Colorectal), GSE13294 (Jorissen Colorectal) [13], GSE21510 (Sugihara Colorectal) [14], GSE37892 (Olschwang Colorectal) [15] and GSE4459 (Watanabe Colorectal) [16] were analyzed in this study. The RNA-sequencing files and microarray files were downloaded from TCGA and GEO databases, respectively.

### Tissue samples and clinical data collection

Two independent cohorts including 308 CRC patients were enrolled for this study. In cohort 1, 126 paired CRC tissues and adjacent nontumor tissues were gathered from patients during operation at Affiliated Nanjing First Hospital of Nanjing Medical University (Nanjing, China). In cohort 2, 182 paraffin-embedded CRC specimens were collected from the Department of Pathology in Nanjing First Hospital. The patient information was listed in Table 1. This study was approved by the ethics committee on Human Research of the Nanjing First Hospital and written informed consent was obtained from all patients.

**Table 1:** The clinic-pathological factors of CRC patients

Characteristics	Number of cases *(Cohort 1)*	SATB2-AS1 expression		*P* value^a^	Number of cases *(Cohort 2)*	SATB2-AS1 expression		*P* value^a^
		Low (n= 63)	High (n= 63)			Low (n= 91)	High (n= 91)	
Age (year)								
≤ 60	61	31	30	0.859	96	48	48	0.882
> 60	65	32	33		86	43	43	
Gender								
Female	54	27	27	1.000	88	38	50	0.102
Male	72	36	36		94	53	41	
Tumor invasion depth								
T1-2	63	21	42	**< 0.001**	81	32	49	**0.017**
T3-4	63	42	21		101	59	42	
Lymph node metastasis								
N0	70	24	46	**< 0.001**	106	39	67	**< 0.001**
N1+N2	56	39	17		76	52	24	
Distant metastasis								
M0	111	50	61	**0.002**	157	70	87	**< 0.001**
M1+M2	15	13	2		25	21	4	
TNM stage								
I+II	67	22	45	**< 0.001**	97	33	64	**< 0.001**
III+III	59	41	18		85	58	27	

^a^Statistical significant results (in bold)

### Cell lines

The human CRC cell lines (HCT-116, HT-29, SW-620, HCT-8, SW-480, and DLD-1) were purchased from the American Type Culture Collection and the human normal colorectal epithelial cell line NCM460 was obtained from INCELL (San Antonio, USA). All cells had been authenticated through short tandem repeat profiling. SW-480, SW-620, DLD-1 and HT-29 cells were cultured in Dulbecco’s modified Eagle’s medium (DMEM) with 10% fetal bovine serum. HCT-116, HCT-8 and NCM460 were maintained in RPMI-1640 with 10% fetal bovine serum. The cells were cultured in a humidified atmosphere of 5% CO_2_ at 37 °C. All cells were routinely tested and found negative for mycoplasma.

### Plasmid construction and cell transfection

The full-length complementary cDNAs of human SATB2, WDR5 and GADD45A were synthesized and cloned into the expression vector pcDNA3.1 (Invitrogen, China). The small hairpin RNA (shRNA) of SATB2-AS1 was synthesized and cloned into the pGLVH1/GFP/Puro vector (GenePharma, China). SATB2-AS1 siRNAs were designed and synthesized by Ambion (USA). The plasmid vectors and siRNAs were transfected into CRC cells using Lipofectamine 3000 (Invitrogen, USA) according to the protocol. All siRNA and shRNA sequences are listed in Additional file 1: Table S1.

### In vivo metastasis assay

Five-week-old male BALB/c nude mice were maintained and handled according to instructions approved by the Animal Care Committee of Nanjing Medical College. The indicated stably transfected HCT-116 cells (3 × 10^6^/0.2 ml PBS) were tail-vein injected into the nude mice. All mice were sacrificed 80 days later, and the lungs were surgically dissected. The lung tissues were embedded in paraffin for hematoxylin and eosin (HE) staining and statistical analysis of the number of tumor nodules. During this period, computed tomographic (CT) scans were performed on the lungs to observe the metastatic conditions.

### Gene set enrichment analysis (GSEA) and single sample gene set enrichment analysis (ssGSEA)

The GSEA was employed to identify gene sets correlated with SATB2-AS1 in CRC. Gene expression profiles of CRC were obtained from the TCGA dataset. CRC samples were divided into a high expression group and a low expression group according to the expression of SATB2-A1 (the top 25% samples grouped as high expression and the bottom 25% samples were grouped as low expression). The GSEA v3.0 tool was used to explore the distribution of members of the gene sets from the MSigDB database [17]. If most members in a gene set were positively or negatively correlated with the SATB2-AS1 expression, the set was termed associated with SATB2-AS1.

The ssGSEA was used to identify the abundance of tumor immune infiltrating cells in CRC tissues. Marker genes of 24 immune cells were obtained from the data set of Bindea et al. [18]. A deconvolution analysis was conducted using the R package GSVA to estimate immune cell populations according to a previous report [19].

### RNA pull-down assay

SATB2-AS1 full-length sense, antisense, and serial deletion sequences were in vitro transcribed with Biotin RNA Labeling Mix and T7 RNA polymerase (Roche, Switzerland), and purified with the RNeasy Mini Kit (Qiagen, USA) according to the manufacturer’s instructions. Biotin labeled SATB2-AS1 was incubated with total cell lysates of CRC and eluted proteins were purified and detected by silver staining or western blot.

### Chromatin immunoprecipitation assay

The ChIP assay was performed using the ChIP Assay Kit (Beyotime, China) following the manufacturer’s guidelines. Briefly, CRC cells were cross-linked with 1% formaldehyde solution for 10 min at room temperature and quenched with 125 mM glycine. DNA fragments ranging from 200 to 500 bp were obtained by ultrasonication. Then the lysate was immunoprecipitated with anti-H3K4me3 or IgG antibodies. Immunoprecipitated DNAs were analyzed by qRT-PCR. The ChIP primers are listed in Additional file 1: Table S2. The antibodies used in ChIP are listed in Additional file 1: Table S3.

### Chromatin isolation by RNA purification

The ChIRP assay was performed using the Magna ChIRP RNA Interactome Kit (Millipore, USA) following the manufacturer’s guidelines. Briefly, a total of 1 × 10^7^ cells was lysed in complete lysis buffer for each reaction, and the DNA was then sheared into small fragments through sonication. Then the lysate was incubated with biotin-labeled probes that could hybridize with SATB2-AS1. We divided probes into the ‘odd’ group and the ‘even’ group according to their orders, and probes targeting LacZ were selected as nonspecific controls. Finally, the probes were extracted by streptavidin magnetic beads, and the combined DNA was isolated for qRT-PCR. Probe information is listed in Additional file 1: Table S2.

### Statistical analysis

All statistical analyses were performed using SPSS 18.0 (SPSS, USA) and GraphPad Prism 6 (GraphPad, USA) software. A chi-square test was employed to analyze the distribution differences of the variables. Student’s t-test was conducted to analyze the differences in gene expression. Univariate Cox proportional hazards regression was used to find potential prognosis associated factors. Estimation of survival difference was performed using the Kaplan-Meier method and log-rank test. For in vitro and in vivo experiments, the t-test or analysis of variance was used to evaluate the difference between different groups. The Pearson correlation coefficient was used to evaluate the correlation of expression. All *P*-values were two-sided, and *P* <  0.05 was statistically significant. All data are presented as the mean ± standard deviation (SD) from at least three independent replicates.

A complete description of the methods, including RNA isolation and qRT-PCR, 5′ and 3′ rapid amplification of cDNA ends analysis (RACE), enzyme-linked immunosorbent assay (ELISA), protein extraction and western blot, immunohistochemistry (IHC), RNA ISH, immunofluorescence (IF), RNA fluorescence in situ hybridization (FISH), subcellular fractionation location, transwell migration and matrigel invasion assays, wound healing assay and RIP assay are available in Additional file 2: Supplementary materials and methods.

## Results

### Colorectal tissue-specific lncRNA SATB2-AS1 is downregulated in CRC tissues and predicts a good prognosis

**Fig. 1:**
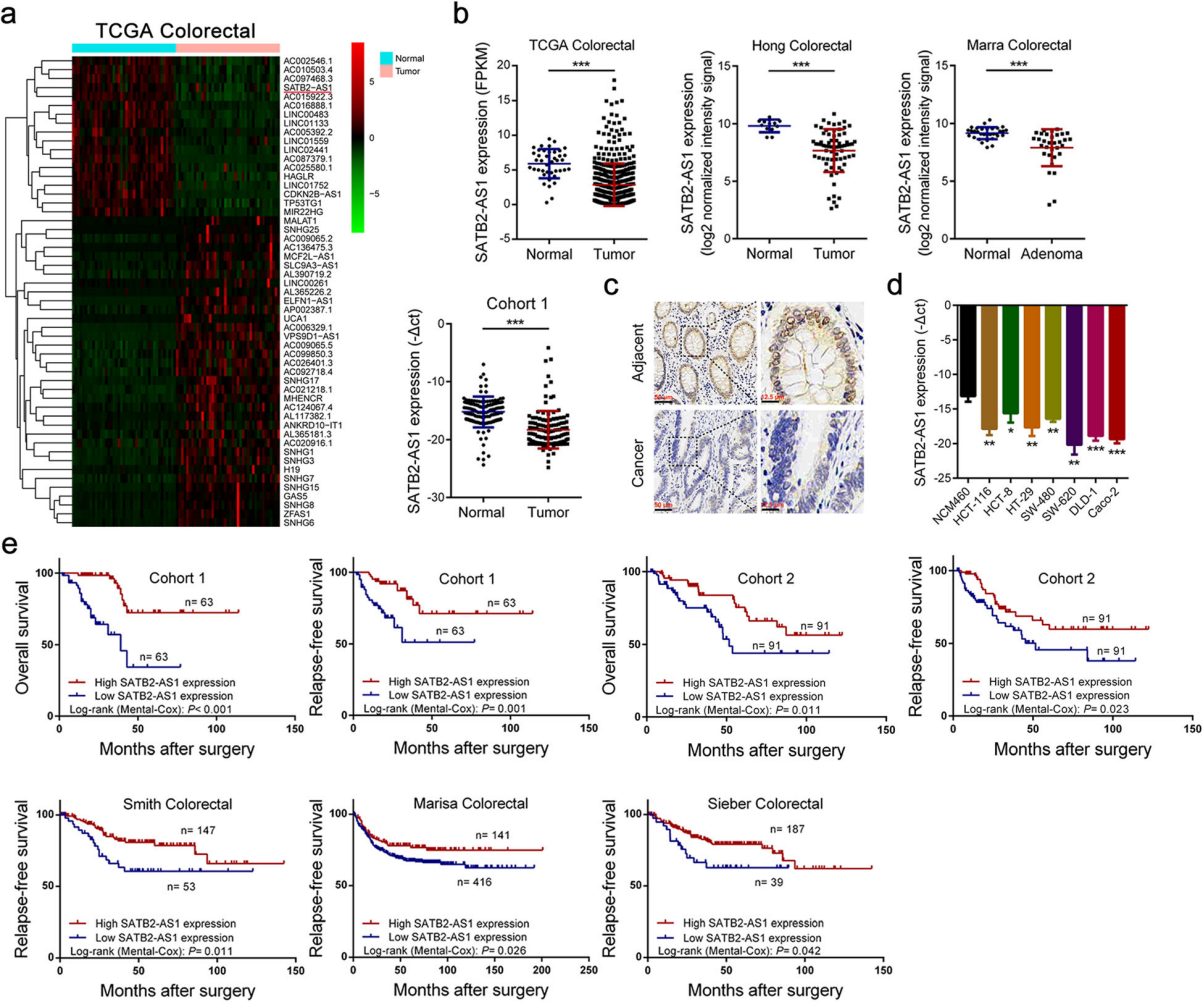
SATB2-AS1 expression is downregulated in CRC and is correlated with prognosis. **a**, Hierarchical cluster heat map of differentially expressed lncRNAs in CRC and corresponding normal tissues generated from RNA sequencing data from the TCGA database. Red in the heat map denotes upregulation; green denotes downregulation. The red line indicates SATB2-AS1. **b**, Expression of SATB2-AS1 in the TCGA CRC cohort, Hong CRC cohort, Marra CRC cohort and our CRC cohort 1. **c**, Representative images of SATB2-AS1 expression in CRC and adjacent colorectal tissues detected by ISH in our CRC cohort 1. **d**, SATB2-AS1 expression in CRC cell lines (HCT-116, HCT-8, HT-29, SW-480, SW-620, DLD-1 and Caco-2) compared with the normal colorectal epithelial cell NCM460 detected by qRT-PCR. **e**, Survival was analyzed and compared between patients with high and low levels of SATB2-AS1 expression in tumors in the corresponding CRC cohort using the Kaplan-Meier method. *, *P* < 0.05; **, *P* < 0.01 and ***, *P* < 0.001

To discover the lncRNAs that was involved in CRC, we first analyzed the lncRNA expression profiles of CRC in two independent cohorts. We identified a series of abnormally expressed lncRNAs in CRC and found that SATB2-AS1 was significantly decreased in both cohorts (Fig. 1a, Additional file 3: Figure S1a). This result was confirmed by qRT-PCR analysis of our CRC cohort and data analysis of other independent cohorts (Fig. 1b). In addition, by analyzing data in cBioPortal [20], we found that SATB2-AS1 was specifically expressed in colorectal tissues, and its expression in colorectal tissues was remarkably higher than that in other tissues (Additional file 3: Figure S1b). Therefore, we speculated that it may play an important role in the development and homeostasis of colorectal tissue. SATB2-AS1 is located on chromosome 2q33. We used rapid the amplification of cDNA ends method to obtain the whole sequence of SATB2-AS1 in HCT-116 cells (Additional file 3: Figure S1c). The conservation analysis of SATB2-AS1 was conducted in the UCSC Genome Browser database [21] (Additional file 3: Figure S1d). In addition, we used the Coding Potential Calculator tool [22] and an in vitro translation assay to assess the protein-coding potential of SATB2-AS1 and found that it was a non-coding transcript (Additional file 3: Figure S1e and Figure S1f). We designed and synthesized in situ hybridization probes of SATB2-AS1 and detected its expression by ISH in tissues. Consistent with the results of qRT-PCR, SATB2-AS1 expression was significantly lower in CRC than in adjacent normal tissues (Fig. 1c). We also detected SATB2-AS1 expression in cell lines, as shown in Fig. 1d, and its expression in the normal colorectal epithelial cell line NCM460 was significantly higher than that in CRC cells (HCT-29, DLD-1, SW-620, HCT-8, HCT-116, Caco-2 and SW-480). We next analyzed the relationship between SATB2-AS1 expression and the clinicopathologic features of CRC. We divided the enrolled patients into two groups based on SATB2-AS1 expression. A chi-square test was used to analyze the distribution differences of different clinical features between the two groups. As shown in Table 1, SATB2-AS1 expression was correlated with tumor invasion depth (*P* <  0.001 in cohort 1, *P* = 0.017 in cohort 2), lymph node metastasis (P <  0.001 in cohort 1, P <  0.001 in cohort 2), distant metastasis (*P* = 0.002 in cohort 1, *P* <  0.001 in cohort 2) and TNM stage (*P* <  0.001 in cohort 1, P <  0.001 in cohort 2). Additionally, survival analysis using the Kaplan-Meier method demonstrated that a lower SATB2-AS1 level was associated with poor overall survival and poor relapse-free survival in our two CRC cohorts and three other independent cohorts (Fig. 1e). In addition, univariate regression analyses of our cohorts demonstrated that SATB2-AS1 expression was an independent predictor for predicting the overall survival of CRC patients (Hazard ratio [HR] = 0.28, 95% confidence interval [CI] = 0.10–0.78, *P* = 0.015, Additional file 3: Figure S1g).

### SATB2-AS1 inhibits CRC metastasis, and its expression correlates with intratumoral immune cell abundance in CRC tissues

**Fig. 2:**
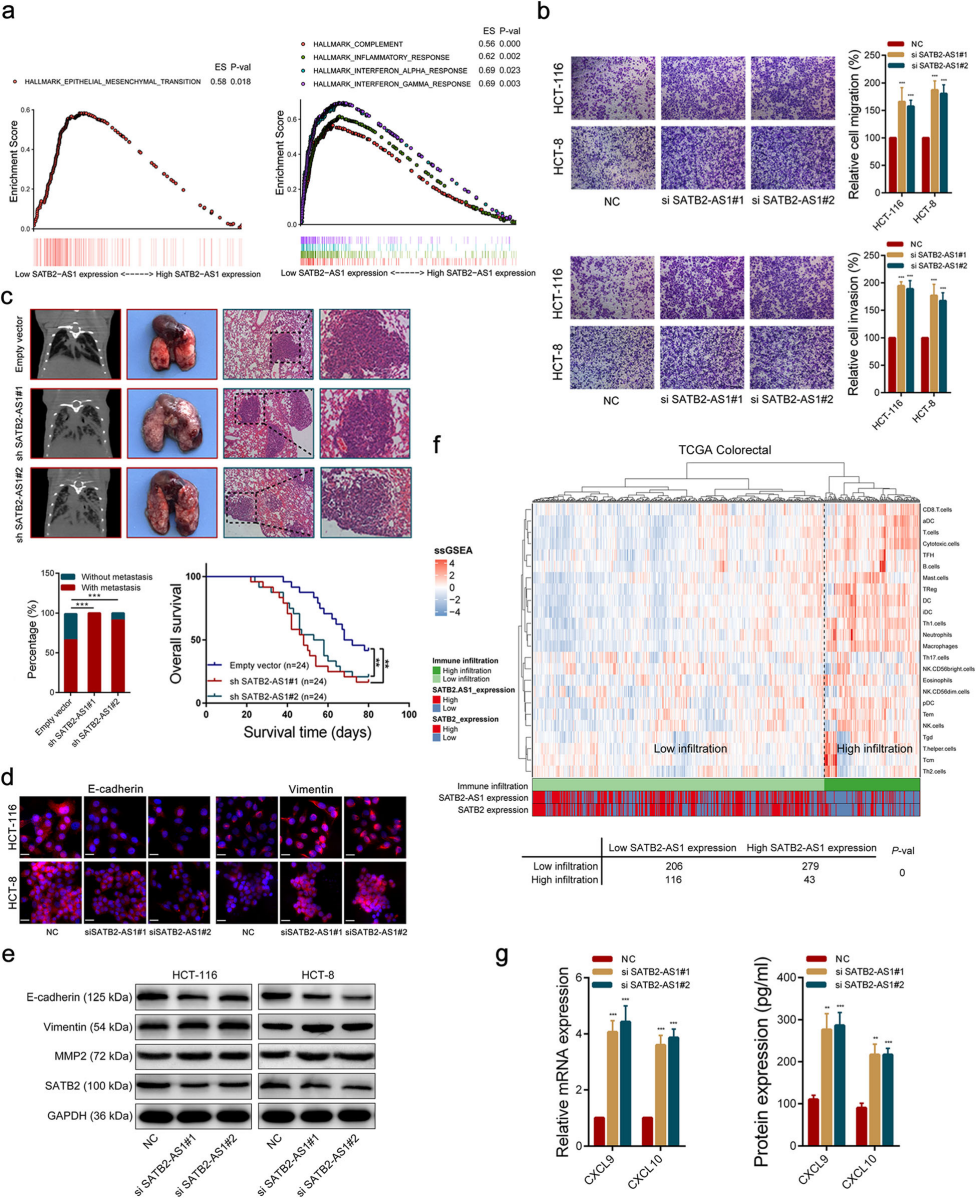
SATB2-AS1 inhibits CRC metastasis and regulates the immune response of CRC. **a,** GSEA results were plotted to visualize the correlation between the expression of SATB2-AS1 and genes related to the EMT (epithelial-mesenchymal transition, left) and immune response (right). **b,** Transwell assays were used to determine the invasion and migration abilities of SATB2-AS1 siRNAs-transfected CRC cells. **c,** Upper panel, representative CT scans, images of the gross lesion in lung tissues and HE staining of metastatic nodules in the lungs from the different group. Lower panel, summarized data on metastatic rates and survival analysis was performed in mice bearing CRC transfected with the indicated cells. **d,** The E-cadherin and Vimentin protein levels were detected by immunofluorescence after SATB2-AS1 knockdown. **e,** SATB2 and metastasis-related proteins were detected by western blot after SATB2-AS1 knockdown. **f,** Upper panel, unsupervised clustering of CRC patients from the TCGA cohort using single-sample gene set enrichment analysis scores from 24 immune cell types. SATB2-AS1 and SATB2 expression were annotated in the lower panel. Hierarchical clustering was performed with Euclidean distance and Ward linkage. Two distinct immune infiltration clusters, here termed high infiltration and low infiltration, were defined. Lower panel, chi-square test results showed differences in sample distribution. **g,** CXCL9 and CXCL10 were detected by qRT-PCR (left) and ELISA (right) after SATB2-AS1 knockdown in HCT-116 cells. Scale bar = 20 μm. **, *P* < 0.01 and ***, *P* < 0.001

To elucidate the biological pathways in which SATB2-AS1 is involved in CRC pathogenesis, based on its expression levels, we performed gene set enrichment analysis using transcriptome data of CRC in the TCGA dataset. The GSEA results indicated that the gene signatures of HALLMARK_EPITHELIAL_MESENCHYMAL_TRANSITION, HALLMARK_INFLAMMATORY_RESPONSE, HALLMARK_COMPLEMENT, HALLMARK_INTERFERON_GAMMA_RESPONSE and HALLMARK_INTERFERON_ALPHA_RESPONSE were enriched in patients with low SATB2-AS1 expression (Fig. 2a). These data suggest that SATB2-AS1 may be a vital modulator in CRC metastasis and the immune response. To dissect the effect of SATB2-AS1 in CRC, we knockdown SATB2-AS1 using two chemically modified siRNAs in HCT-116 and HCT-8 cells, and the efficiencies of interference were confirmed by qRT-PCR (Additional file 3: Figure S2a). Transwell assays demonstrated that SATB2-AS1 knockdown significantly increased CRC cell migration and invasion ability (Fig. 2b). Wound healing assays showed that CRC cells with SATB2-AS1 knockdown underwent faster scratch wound closure than the negative control (NC) cells (Additional file 3: Figure S2b). To further evaluate the anti-metastatic effect of SATB2-AS1 in vivo, HCT-116 cells stably transfected with shRNA targeting SATB2-AS1 were injected into nude mice from the tail vein. The efficiencies of sh-RNAs were evaluated by qRT-PCR (Additional file 3: Figure S2c). The mouse lung metastatic models showed that silencing SATB2-AS1 significantly promoted lung metastasis. Compared with the empty vector group, more metastatic lesions were observed by CT in mouse lungs in the two SATB2-AS knockdown groups. Mice that received SATB2-AS1 knockdown HCT-116 cells had a shorter overall survival than mice that were inoculated with empty vector transfected HCT-116 cells. In addition, there were more metastatic foci in the lungs of nude mice in the SATB2-AS1 knockdown groups than in the empty vector group (Fig. 2c). Moreover, IF results showed that silencing SATB2-AS1 decreased E-cadherin expression and increased Vimentin expression, which were consistent with western blot results. The western blot results also showed that another metastasis-related protein, MMP9, was upregulated in SATB2-AS1 knockdown CRC cells (Fig. 2d, e). Through correlation analysis of transcriptome data in TCGA, we found that the expression level of SATB2-AS1 was positively correlated with the E-cadherin RNA level (r = 0.33, *p* <  0.001) and negatively correlated with the TGF beta 1 (r = − 0.26, p <  0.001) and Vimentin RNA levels (r = − 0.18, p <  0.001) in CRC tissues (Additional file 3: Figure S2e). These results suggest that SATB2-AS1 can inhibit the metastasis of CRC cells.

The immune response and immune environment have an important influence on tumor initiation, progression and therapy. The GSEA results indicated the expression of SATB2-AS1 was correlated with various immune responses. We then used ssGSEA to quantify tumor-infiltrating immune cells in CRC tissues from transcriptomics data. Based on the number of infiltrating immune cells in the tissues, we divided the CRC samples in the TCGA cohort into the ‘high infiltration’ group and the ‘low infiltration’ group. We found that the SATB2-AS1 expression of most samples in the ‘high infiltration’ group was lower than the median value of all samples, and the chi-square test results showed that the difference in sample distribution was statistically significant (Fig. 2f). In addition, we found that the expression of the TH1-type chemokines CXCL9 and CXCL10, which could be induced by interferon gamma (IFN-γ) and mediate effector T-cell trafficking, was increased when SATB2-AS1 was knockdown in CRC cells (Fig. 2g, Additional file 3: Figure S2d). Correlation analysis showed that SATB2-AS1 expression was negatively correlated with IFN-γ (IFNG, r = − 0.20, p <  0.001)), CXCL9 (r = − 0.20, p <  0.001) and CXCL10 (r = − 0.19, *p* < 0.001) RNA levels in CRC tissues (Additional file 3: Figure S2e). Altogether, these results indicate that SATB2-AS1 may be a mediator of the CRC immune response and a potential target for CRC immunotherapy.

### SATB2-AS1 exerts its biological roles by regulating SATB2 in CRC

**Fig. 3:**
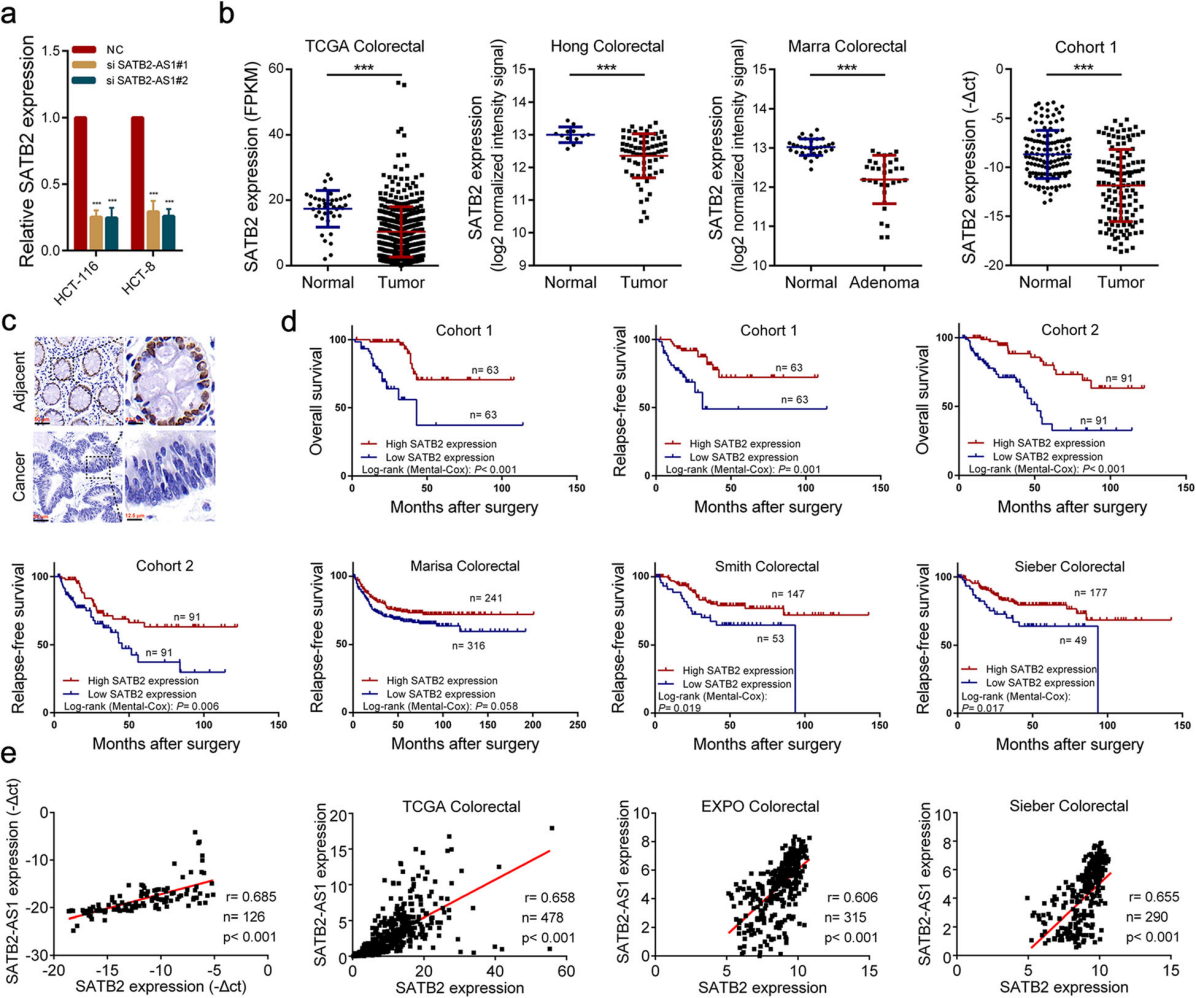
SATB2 is down-regulated in CRC and regulated by SATB2-AS1. **a**, SATB2 was detected by qRT-PCR after SATB2-AS1 knockdown in CRC cells. **b**, Expression of SATB2 in the TCGA CRC cohort, Hong CRC cohort, Marra CRC cohort and our CRC cohort 1. **c**, Representative images of SATB2 expression in CRC and adjacent colorectal tissues detected by IHC in our CRC cohort 1. **d**, Survival was analyzed and compared between patients with high and low SATB2 expression in indicated CRC cohorts by Kaplan-Meier method. **e**, Correlation analysis of the expression of SATB2-AS1 and SATB2 in indicated CRC cohorts. ***, *P* < 0.001

Many antisense lncRNAs work by regulating the expression of their neighboring gene. Consistent with speculation, silencing SATB2-AS1 obviously reduced the RNA and protein levels of its cognate sense gene SATB2 (Figs. 2e, 3a). By analyzing the transcriptome and microarray data, we found that SATB2 expression was significantly reduced in CRC tissues. Our qRT-PCR and IHC results confirmed the change (Fig. 3b, c). In addition, by analyzing the transcriptome data in TCGA, we found that the expression of SATB2 in colorectal tissues was obviously higher than that in other tissues like SATB2-AS1 (Additional file 3: Figure S3a). Survival analysis in five independent CRC cohorts indicated that low expression of SATB2-AS1 was associated with shorter overall survival and relapse-free survival (Fig. 3d). Correlation analysis showed that SATB2-AS1 expression was significantly positively correlated with SATB2 in CRC tissues (Fig. 3e, Additional file 3: Figure S3b). In conclusion, SATB2 may play an important role in CRC, and SATB2-AS1 can regulate SATB2 expression in CRC.

**Fig. 4:**
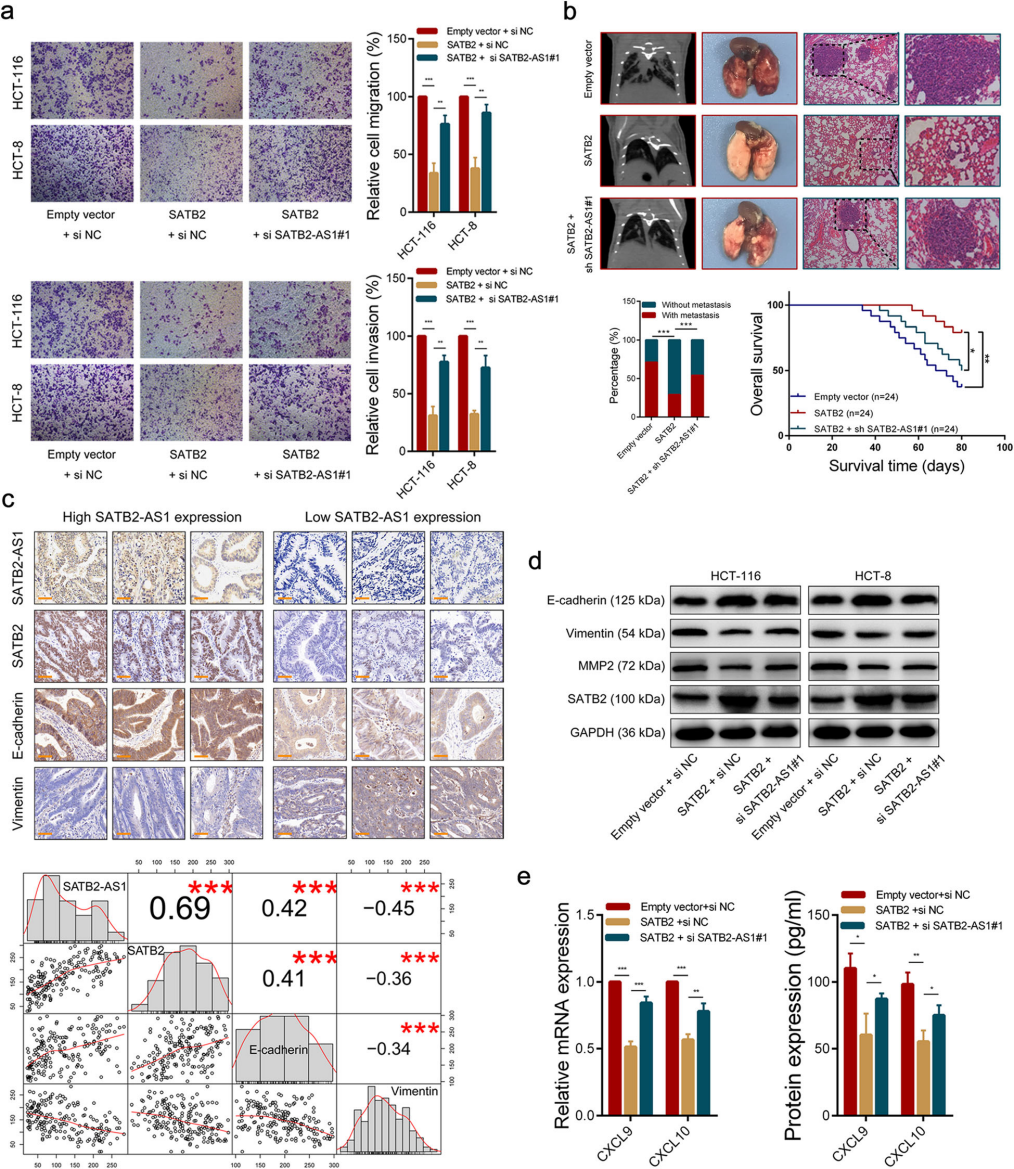
SATB2-AS1 exerts its biological roles by regulating SATB2. **a**, Transwell assays were used to determine the invasion and migration abilities of CRC cells with the indicated treatment. **b**, Upper panel, representative CT scans, images of the gross lesion in lung tissues and HE staining of metastatic nodules in the lungs from the indicated group. Lower panel, summarized data on metastatic rates and survival analysis was performed in mice bearing CRC transfected with corresponding cells. **c**, Upper panel, representative images of SATB2-AS1 expression in CRC tissues detected by ISH and SATB2, E-cadherin, and Vimentin expression in CRC tissues detected by IHC in our CRC cohort 2. Lower panel, scatter plot showing the expression relationship among SATB2-AS1, SATB2, E-cadherin and Vimentin in our CRC cohort 2. The upper right squares show the Pearson correlations among them. **d**, SATB2, E-cadherin, Vimentin and MMP2 were detected by western blot in CRC cells with the indicated treatment. **e**, CXCL9 and CXCL10 were detected by qRT-PCR (left) and western blot (right) in HCT-116 cells with the indicated treatment. Scale bar = 20 μm. *, *P* < 0.05; **, *P* < 0.01 and ***, *P* < 0.001

To investigate whether SATB2-AS1 plays a role in CRC by regulating SATB2, we conducted a series of rescue experiments. Transwell assay results indicated that SATB2 overexpression could inhibit the migration and invasion ability of CRC cells, and knockdown of SATB2-AS1 in SATB2-overexpressing cells could partially alleviate the inhibition (Fig. 4a). Wound healing assay results showed that silencing SATB2-AS1 could partly abolish the deceleration of scratch wound closure in CRC cells caused by SATB2 overexpression (Additional file 3: Figure S4a). Mice inoculated with SATB2-overexpressing HCT-116 cells had a longer overall survival time and lower metastasis rate than mice that received empty vector-expressing tumor cells, and there were fewer metastatic foci in the lungs of nude mice after the injection of SATB2-overexpressing HCT-116 cells when compared with control groups. In addition, silencing of SATB2-AS1 partially restored the metastasis capacity inhibited by SATB2 overexpression (Fig. 4b). In line with our speculation, the expression of SATB2 decreased after the transfection of SATB2-AS1 sh-RNAs into SATB2-overexpressing HCT-116 cells (Additional file 3: Figure S4b). Western blot results demonstrated that the protein level of the epithelial marker E-cadherin was significantly increased in SATB2-overexpressing CRC cells and that this increase could be partly impaired by SATB2-AS1 silencing. Conversely, the expression of the mesenchymal markers Vimentin and MMP-9 were decreased in SATB2-overexpressing CRC cells and their decreases could be partly rescued by SATB2-AS1 silencing (Fig. 4d). The same results were observed in immunofluorescence assays (Additional file 3: Figure S4c). Consistently, ISH and IHC staining analysis of CRC tissues in Cohort 2 (*n* = 182) for SATB2-AS1, SATB2 and EMT markers indicated that patients with low SATB2-AS1 expression displayed lower SATB2 and E-cadherin expression and higher Vimentin expression compared with patients with high SATB2-AS1 expression. Additionally, SATB2-AS1 expression levels were positively correlated with SATB2 (r = 0.69, *p* < 0.001) and E-cadherin (r = 0.421, p < 0.001) expression, and negatively correlated with the expression levels of Vimentin (r = − 0.45, p < 0.001, Fig. 4c). In addition, SATB2 overexpression suppressed CXCL9 and CXCL10 expression in CRC cells and SATB2-AS1 knockdown partially alleviated the decrease of CXCL9 and CXCL10 expression caused by SATB2 (Fig. 4e, Additional file 3: Figure S4d). Collectivity, these data suggest that SATB2-AS1 suppresses CRC metastasis and regulates the CRC immune response by regulating SATB2 expression.

### SATB2-AS1 directly binds to WDR5 and GADD45A

**Fig. 5:**
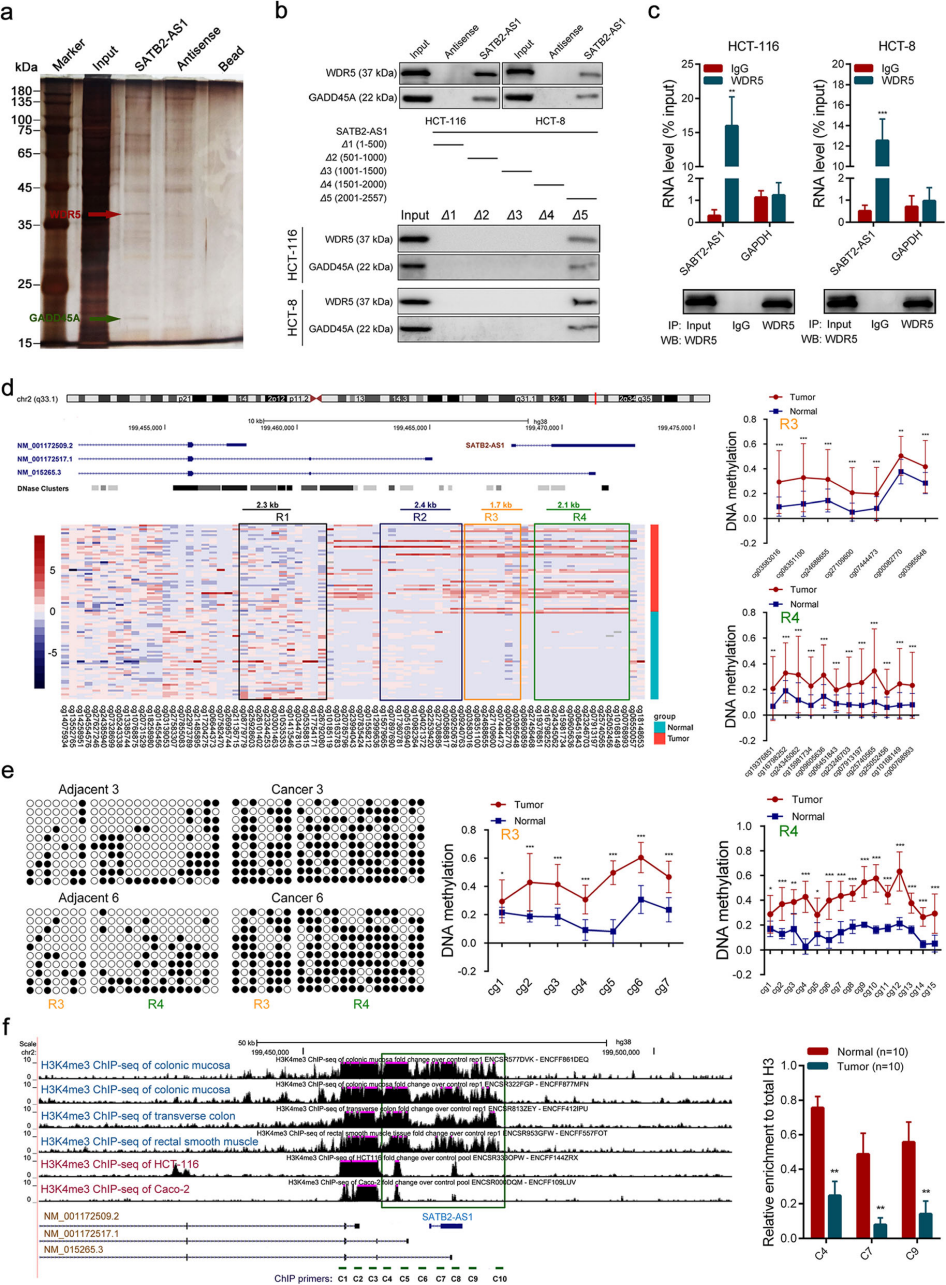
SATB2-AS1 directly binds to WDR5 and GADD45A, and downregulation of SATB2 in CRC is due to high DNA methylation and loss of histone H3K4me3. **a**, Representative image of silver-stained PAGE gels showing separated proteins that were pulled down using biotin-labeled SATB2-AS1. A red arrow indicates WDR5, and a green arrow indicates GADD45A. **b**, Upper panel, western blot of the proteins from antisense SATB2-AS1 and SATB2-AS1 pull-down assays. Lower panel, western blot of WDR5 and GADD45A in samples pulled down by full-length or truncated SATB2-AS1 (Δ1: 1–500, Δ2: 501–1000, Δ3: 1001-1500, Δ4: 1501–2000, Δ5: 2001–2557). **c**, RNA immunoprecipitation with an anti-WDR5 antibody was used to assess endogenous WDR5 binding to RNA in CRC cells, IgG was used as the control. SATB2-AS1 and GAPDH levels were determined by qRT-PCR and are presented as fold enrichment in WDR5 relative to input. The RIP efficiency of WDR5 protein was detected by western blot. **d**, Left panel, DNA methylation analysis of SATB2 in CRC and corresponding normal tissues generated from the 450 k DNA methylation data in the TCGA database. Red in the heatmap denotes upregulation of methylation level; blue denotes downregulation. Each square represents a single CpG site, and each row represents a tissue sample. A scheme representing the SATB2 and SATB2-AS1 locus including the sites of each transcript, is displayed above. Right panel, statistical analysis of differences in methylation levels between tumors and adjacent normal tissues in the R3 and R4 regions. **e**, Bisulfite sequencing was performed to detect CpG island methylation statuses in R3 and R4 regions in ten paired CRC tissues and adjacent normal tissues. Representative images and corresponding statistical plots were presented. Black solid and white hollow circles represent methylated and unmethylated CpG sites, respectively. **f**, Left panel, analysis of H3K4me3 ChIP-seq data of colorectal tissues and CRC cells in the SATB2 locus. Right panel, the relative H3K4me3 levels of the SATB2 promoter in ten CRC tissues and matched adjacent colorectal tissues were evaluated by ChIP-qPCR. *, *P* < 0.05; **, *P* < 0.01 and ***, *P* < 0.001

Next, we explored the potential molecular mechanisms of SATB2-AS1 in the expression of SATB2. Given that the function of a lncRNA is related to its subcellular localization, we first identified the subcellular distribution of SATB2-AS1 through fluorescence in situ hybridization and subcellular fractionation assays. The results demonstrated that SATB2-AS1 was mostly expressed in the cell nucleus (Additional file 3: Figure S5a and Figure S5b). Numerous nuclear lncRNAs have been reported to interact with chromatin-modulating proteins, facilitating their recruitment to chromatin, thereby controlling transcriptional activity. We then identified intracellular SATB2-AS1-interacting proteins using an unbiased approach. Biotinylated SATB2-AS1 was incubated with total protein extracts from HCT-116 cells and pulled down by streptavidin magnetic beads. The binding proteins were analyzed using silver staining. There were two specific bands in the SATB2-AS1 pull-down samples, which were excised and subjected to mass spectrometry analysis. Based on the functional annotation of MS predicted proteins and a literature review, WDR5, a core subunit of the human histone H3K4 methyltransferase complex, on the band with a molecular weight between 35 kDa and 40 kDa, and GADD45A, a regulator of DNA demethylation, on the other evident band with a molecular weight between 15 kDa and 25 kDa, were selected as potent SATB2-AS1-interacting proteins (Fig. 5a). Western blot assays further validated the binding of SATB2-AS1 to WDR5 and GADD45A using the retrieved proteins from the RNA pull-down assays of CRC cells. To identify the SATB2-AS1-interacting region of WDR5 and GADD45A, we constructed and biotinylated five fragments of SATB2-AS1 (Δ1:1–500, Δ2:501–1000, Δ3:1001–1500, Δ4:1501–2000, Δ5:2001–2557) and used them to conduct the pull-down assay with CRC cell lysates. The results showed that the 3′ fragment of SATB2-AS1 mediated the interaction with both WDR5 and GADD45A (Fig. 5b). In addition, RIP assays performed with an anti-WDR5 antibody also revealed the interaction between WDR5 and SATB2-AS1 (Fig. 5c). In summary, these data indicate that SATB2-AS1 physically interacts with WDR5 and GADD45A in CRC cells.

### Low expression of SATB2 in CRC is due to high DNA methylation and loss of histone H3K4me3 in the promoter region

Because the SATB2-AS1-binding proteins WDR5 and GADD45A are vital chromatin-modulating factors, we hypothesized that SATB2-AS1 may promote SATB2 expression by recruiting WDR5 and GADD45A to the SATB2 promoter region and increasing its histone H3K4me3 enrichment and activating its DNA demethylation [23, 24]. Therefore, we first explored whether the decreased expression of SATB2 in CRC tissues was caused by increased DNA methylation and reduced histone H3K4me3 enrichment in its promoter region. According to the information in the NCBI Reference Sequences database, SATB2 has three reviewed transcripts (NM_001172509, NM_001172517, NM_015265). We detected these three transcripts in CRC cells by PCR. The results displayed that all three transcripts were expressed in CRC cells, and the abundance of the transcript NM_001172517 was the highest (Additional file 3: Figure S5c). Through analyzing the 450 k DNA methylation data of the TCGA CRC cohort, we found the DNA methylation level in some regions of the SATB2 promoter was significantly different between CRC and adjacent normal tissues. As shown in Fig. 5d, the DNA methylation degree of the R3 and R4 regions was significantly higher in CRC. However, there was no significant difference in the R1 and R2 regions between CRC and adjacent tissues (Additional file 3: Figure S5d). To further confirm the DNA methylation status of the SATB2 promoter region, we detected the methylation levels of the R1, R3 and R4 regions in 10 pairs of CRC and adjacent normal tissues using a BSP assay. The results were consistent with the data in TCGA (Fig. 5e, Additional file 3: Figure S5e and Figure S5f). We then explored the level of H3K4me3 enrichment in the SATB2 promoter region in colorectal tissues and CRC tissues (cells). By analyzing ChIP-Seq data of H3K4me3 in the Encyclopedia of DNA Elements (ENCODE) database [25], we found that the enrichment of H3K4me3 in the SATB2 promoter region was significantly lower in CRC cells than in normal colorectal tissues (Fig. 5f, left panel). In addition, the enrichment of H3K4me3 in the SATB2 promoter region (C4, C7, C9 locus) was detected by ChIP-qPCR in 10 pairs of CRC and adjacent tissues, which was consistent with the ChIP-seq data, the enrichment of H3K4me3 in the SATB2 promoter region was significantly reduced in the CRC tissues (Fig. 5f, right panel). Furthermore, we treated HCT-116 cells with the DNA Methyltransferase inhibitor Azacitidine (5 uM, 48 h) and we found both RNA and protein levels of SATB2 were increased significantly after the treatment. We also treated NCM460 cells with the WDR5 antagonist OICR-9429 (5 uM, 48 h), and we found both RNA and protein levels of SATB2 were significantly decreased (Additional file 3: Figure S5g and Figure S5h). Together, these results indicate that DNA hypermethylation and histone H3K4me3 loss in the promoter region resulted in a decrease of SATB2 expression in CRC.

### SATB2-AS1 modulates DNA demethylation and H3K4me3 enrichment of the SATB2 promoter by recruiting the WDR5 and GADD45A protein

**Fig. 6:**
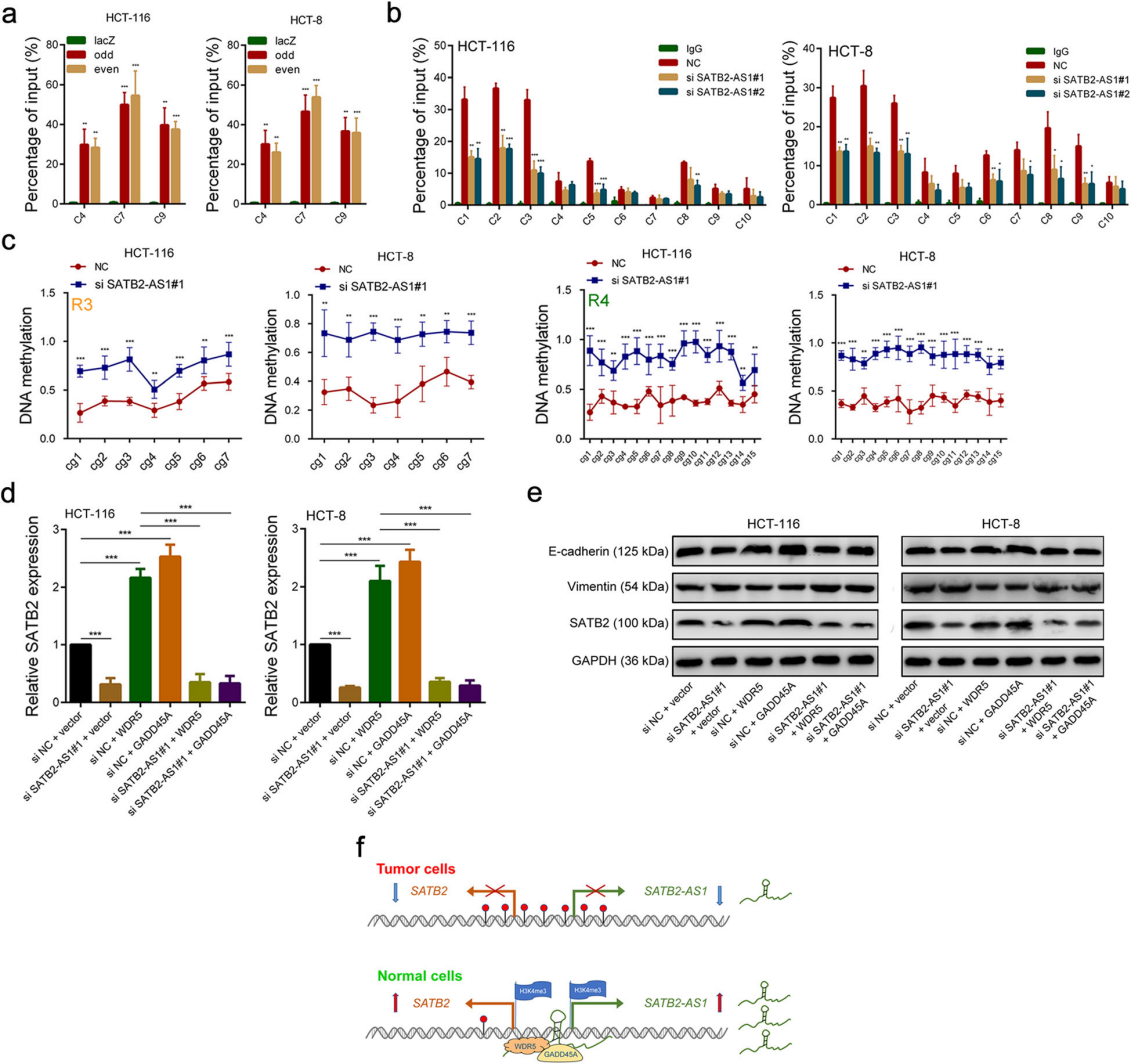
SATB2-AS1 regulates DNA demethylation and H3K4me3 enrichment of the SATB2 promoter by recruiting WDR5 and GADD45A. **a**, SATB2-AS1 targeted probes and negative Laz targeted probes were used for ChIRP assay. Purified DNA and RNA was analyzed by qRT-PCR respectively. ‘Odd’, ‘Even’ and ‘LacZ’ denote the odd- and even-ranked corresponding probes against SATB2-AS1 and probes against bacterial LacZ, respectively. Primer locations of C4, C7 and C9 are shown at the bottom of the Fig. 5f. **b**, ChIP-qPCR assays were performed to detect H3K4me3 occupancy at the SATB2-AS1 promoter region with indicated treatment. Primer locations are shown at the bottom of the Fig. 5f. **c**, Bisulfite sequencing was performed to evaluate the changes of CpG island methylation statuses in R3 and R4 regions in CRC cells after SATB2-AS1 knockdown. **d**, SATB2 levels were determined by qRT-PCR in CRC cells with indicated treatment. **e**, SATB2, E-cadherin and Vimentin expression were detected by western blot in CRC cells with indicated treatment. **f**, Schematic of the proposed mechanism of SATB2-AS1 in CRC. *, *P* < 0.05; **, *P* < 0.01 and ***, *P* < 0.001

Next, we performed ChIRP assays with biotinylated anti-sense oligo probes of SATB2-AS1 and the results indicated that SATB2-AS1 could physiologically associate with the promoter sequences of SATB2 (Fig. 6a, Additional file 3: Figure S6a). ChIP-PCR results showed that the level of H3K4me3 enrichment in the SATB2 promoter region of HCT-116 (C1, C2, C3, C5, C8 locus) and HCT-8 (C1, C2, C3, C6, C7, C8, C9 locus) cells was significantly decreased after SATB2-AS1 knockdown (Fig. 6b). Moreover, the results of BSP experiments demonstrated that the DNA methylation level in the SATB2 promoter region of CRC cells was significantly increased after SATB2-AS1 knockdown (Fig. 6c, Additional file 3: Figure S6b). Besides, we detected the expression of WDR5 and GADD45a in SATB2-AS1 knock down HCT-116 cells and control cells by PCR and found that there were no significant differences in their expression (Additional file 3: Figure S6c). We also detected the expression of WDR5 and GADD45A in 30 pairs of CRC and adjacent tissues. We found WDR5 was significantly highly expressed in CRC tissues and there was no difference in the expression of GADD45 between CRC and adjacent tissues (Additional file 3: Figure S6d). These results indicate that the expression level of SATB2-AS1 does not affect the total expression of WDR5 and GADD45A in CRC, and only affects the amount of WDR5 and GADD45A recruited to the SATB2 promoter region. In addition, we found that overexpression of WDR5 or GADD45A in CRC cells significantly increased the expression of SATB2. As expected, the expression of SATB2 in CRC cells with knocked-down SATB2-AS1 and overexpressed WDR5 or GADD45A did not change compared with those with knocked-down SATB2-AS1 only. Consistent with these results, the epithelial marker E-cadherin was also upregulated in WDR5- or GADD45A-overexpressing CRC cells, and its expression was abolished by SATB2-AS1 knockdown. The mesenchymal marker Vimentin was decreased in WDR5- or GADD45A-overexpressing CRC cells, and its decrease was rescued by SATB2-AS1 knockdown (Fig. 6d, e, Additional file 3: Figure S6e). Collectively, these data demonstrate that SATB2-AS1 promoted SATB2 expression by modulating DNA demethylation and H3K4me3 enrichment of the SATB2 promoter by recruiting WDR5 and GADD45A protein (Fig. 6f).

## Discussion

LncRNAs are important epigenetic regulators that play a vital role in diverse physiological and pathological processes [26, 27]. In tumors, lncRNAs have been reported to be involved in multiple events through tumorigenesis and development, including tumor growth, tumor metastasis, drug resistance, tumor microenvironment regulation, angiogenesis, and cancer stem cell self-renewal [6, 28]. Recent studies have reported that various lncRNAs are abnormally expressed and exert vital functions in CRC. For example, Xiang et al. revealed that the lncRNA CCAT1-L played an oncogene role in CRC by interacting with the MYC locus [29]. Ma et al. demonstrated that the lncRNA CCAL could promote CRC progression by activating the Wnt/β-catenin signaling pathway [30]. Bian et al. found that CCAT1 and CCAT2 could serve as prognostic biomarkers of CRC [31]. These findings indicate that an in-depth understanding of lncRNA roles in CRC may help develop novel diagnosis and treatment strategies. We previously described the roles of SNHG1 and SNHG6 in CRC [32, 33]. However, the role of many other lncRNAs in CRC needs to be explored.

Here, we report a colorectal-specific expressed lncRNA, SATB2-AS1, that inhibits tumor metastasis and regulates the immune response by *cis*-activating SATB2 in CRC. We first identified aberrantly expressed lncRNAs in CRC by analyzing sequencing and microarray data. We revealed that SATB2-AS1 expression was downregulated in CRC tissues and cells, and low SATB2-AS1 expression indicated progression and poor prognosis of CRC. Functional assays demonstrated that SATB2-AS1 could inhibit CRC metastasis both in vitro and in vivo. In addition, SATB2-AS1 could regulate the immune response of CRC, and its expression was associated with the degree of immune cell infiltration in CRC tissues. In terms of mechanism, we found that SATB2-AS1 could promote SATB2 expression by acting as a molecular scaffold of WDR5 and GADD45A in the cell nucleus and recruiting them to the promoter of SATB2.

SATB2-AS1 is an antisense lncRNA located on the opposite strand of SATB2 on chromosome 2q33. We found that the expression of SATB2-AS1 in colorectal tissues was significantly higher than that in other tissues, this indicated that SATB2-AS1 might play a vital role in the development and homeostasis of colorectal tissue. Liu et al. reported that SATB2-AS1 could increase the proliferation of osteosarcoma cells by affecting the expression of SATB2 [34]. However, they did not uncover how SATB2-AS1 regulated SATB2 expression. We first proved that SATB2-AS1 could *cis*-activate SATB2 by mediating H3K4me3 enrichment and DNA demethylation in its promoter region. SATB2-AS1 is a nuclear lncRNA, and many lncRNAs in the nucleus exert an influence on gene expression via histone or DNA modification. We found that SATB2-AS1 could physically interact with WDR5 and GADD45A, two important chromatin-modulating proteins, in CRC cells. WDR5 is a core subunit of the MLL and SET1 histone methyltransferase complexes, which interacts with methylated H3K4 to catalyze Lys4 trimethylation and activates transcription [23, 35]. Previous studies revealed that WDR5 directly binds to hundreds of lncRNAs and various lncRNAs have been reported to serve as a molecular scaffold for WDR5/MLL complexes. Yang et al. reported that a family of lncRNAs that bind WDR5 could increase its stabilization, which promotes MLL complex assembly and methyltransferase activity [36]. Wang et al. found that lncRNA HOTTIP bound WDR5 directly and recruited WDR5/MLL complexes across HOXA, inducing H3K4me3 and gene transcription [37]. In addition, some lncRNAs abnormally expressed in tumors exerted roles by recruiting WDR5 to their targets. Sun et al. reported that lncRNA GClnc1 promoted gastric cancer progression by serving as a modular scaffold of WDR5 and KAT2A to activate SOD2 expression [38]. He et al. reported that lncRNA BLACAT2 epigenetically increased VEGF-C expression by directly binding WDR5 in bladder cancer [39]. GADD45A is a nuclear protein involved in the maintenance of genomic stability and DNA repair and has a key role in DNA demethylation activation. GADD45A interacts with the components of DNA repair complexes, recruits them to corresponding sites and mediates the replacement of methylated cytosines by unmethylated cytosines [24]. Arab et al. reported that lncRNA TARID activated DNA demethylation and expression of the tumor suppressor TCF21 by recruiting GADD45A and GADD45A to bind the R-loop formed by lncRNA [40, 41]. In this study, RNA pull down and RIP data demonstrated that SATB2-AS1 directly binds WDR5 and GADD45A. ChIRP data proved that SATB2-AS1 directly interacted with the promoter of SATB2. Furthermore, we found that SATB2-AS1 promoted SATB2 transcription by activating DNA demethylation and increasing H3K4me3 enrichment of the SATB2 promoter.

SATB2 is a nuclear matrix-associated protein and a vital transcription factor involved in biological development, the regulation of gene expression and chromatin remodeling. The expression of SATB2 is highly tissue specific and it is primarily expressed in glandular cells of the lower gastrointestinal tract and in CRC [42, 43]. Evidence suggests that SATB2 plays diverse and important roles in various cancers [44]. Immunohistochemical analysis of 1882 CRC tissues from nine independent cohorts showed that 85% of tumors were positive for SATB2 and 97% for SATB2 and/or cytokeratin 20, suggesting that SATB2 is a highly sensitive marker to distinguish CRC from other cancers [45]. Zhang et al. reported that SATB2 is a promising biomarker for distinguishing between liver metastases with a CRC origin and other types of adenocarcinoma [46]. In addition, Eberhard et al. reported that high SATB2 expression in CRC was associated with better prognosis and increased the patient’s benefit from chemotherapy and radiation therapy [47]. Magnusson et al. revealed that CRC metastasis has lower SATB2 expression compared with primary CRC, and another study demonstrated that low SATB2 expression is correlated to tumor invasion and metastasis of lymph nodes in CRC [45, 48, 49]. Even so, the role of SATB2 in CRC is not fully understood. Mansour et al. reported that SATB2 suppresses the proliferation, migration and invasion of CRC cells via inactivation of MEK5/ERK5 signaling [50]. Li et al. reported that SATB2 could bind to regulatory elements of the CD133, CD44, MEIS2 and AXIN2 genes, and acts as a negative regulator of stemness in CRC cells [51]. In this study, we found that SATB2 could inhibit CRC metastasis and regulate the immune response of CRC. SATB2 expression was associated with the degree of immune cell infiltration in CRC tissues and could inhibit the expression of the TH1-type chemokines CXCL9 and CXCL10. SATB1, a homologous gene of SATB2, has been reported to act as a critical regulator of tumor immune response [44, 52]. These results strongly suggest that both SATB2-AS1 and SATB2 may be potential immunoregulatory factors and serve as biomarkers and targets for CRC immunotherapy. However, the underlying mechanism of their roles in CRC still needs to be further explored.

In summary, our current work showed that SATB2-AS1 was downregulated in CRC and that low SATB2-AS1 expression was associated with poor survival. SATB2-AS1 inhibits CRC cell metastasis and regulates the immune response of CRC by *cis*-activating SATB2. These data suggest that SATB2-AS1 and SATB2 may be novel biomarkers and promising therapeutic targets in CRC. However, further studies need to be performed to identify the precise molecular mechanism by which SATB2 mediates metastasis and the immune response in CRC.

## Additional files

Additional file 1: **Table S1.** SiRNAs and sh-RNAs sequence. **Table S2.** The list of primers and probes. **Table S3.** Information of antibodies. (ZIP 44 kb)

Additional file 2: Supplementary materials and methods. (DOCX 19 kb)

Additional file 3: **Figure S1.** Colorectal specifically expressed SATB2-AS1 is associated with prognosis in CRC. **Figure S2.** SATB2-AS1 regulates metastasis and immune response of CRC. **Figure S3.** SATB2 is highly expressed in colorectal tissues and correlated with SATB2-AS1 in CRC. **Figure S4.** SATB2-AS1 regulates metastasis and immune response through SATB2 in CRC. **Figure S5.** SATB2-AS1 is mainly distributed in the cell nucleus and down-regulation of SATB2 in CRC is partly due to high DNA methylation. **Figure S6.** SATB2-AS1 binds to the promoter region of SATB2 and recruits WDR5 and GADD45A. (DOCX 4050 kb)

## Data Availability

The datasets used and/or analyzed during the current study are available from the corresponding author on reasonable request.

## Abbreviations

BSP: Bisulfite Sequencing PCR
ChIP: Chromatin immunoprecipitation
ChIRP: Chromatin Isolation by RNA Purification
CI: Confidence interval
CRC: Colorectal cancer
CT: Computed tomographic
DMEM: Dulbecco’s modified Eagle’s medium
ELISA: Enzyme-linked immunosorbent assay
ENCODE: Encyclopedia of DNA Elements
FISH: RNA fluorescence in situ hybridization
GSEA: Gene set enrichment analysis
H3K4me3: Histone H3 lysine 4 tri-methylation
HE: Hematoxylin and eosin
HR: Hazard ratio
IF: Immunofluorescence
IHC: Immunohistochemistry
ISH: in situ hybridization
lncRNAs: long noncoding RNAs
qRT-PCR: quantitative reverse transcription polymerase chain reaction
RACE: 5′ and 3′ rapid amplification of cDNA ends analysis
RIP: RNA immunoprecipitation
SATB2: Special AT-rich binding protein 2
SD: Standard deviation
ssGSEA: Single sample gene set enrichment analysis
