# Supplementary material for: LncRNA SATB2-AS1 inhibits tumor metastasis and affects the tumor immune cell microenvironment in colorectal cancer by regulating SATB2
Source: Mol Cancer. 2019 Sep 6;18:135. doi: 10.1186/s12943-019-1063-6 (PMC6729021; doi:10.1186/s12943-019-1063-6)
Supplement: Supplementary file 1 — Table S1. SiRNAs and sh-RNAs sequence. Table S2. The list of primers and probes. Table S3. Information of antibodies. (ZIP 44 kb) [file 12943_2019_1063_MOESM1_ESM.zip › Table S1.docx]

**Table S1: SiRNAs and sh-RNAs sequence.**

| **siRNAs** |  |  |
| --- | --- | --- |
|  | **sense sequence** | **anti-sense sequence** |
| si-SATB2-AS1#1 | 5’-CCUAUUACAUUUUUCCGGATT-3’ | 5’-UCCGGAAAAAUGUAAUAGGTG-3’ |
| si-SATB2-AS1#2 | 5’-CAGGUAAUGCGCUUCAUUATT-3’ | 5’-UAAUGAAGCGCAUUACCUGTG-3’ |
| **shRNAs** |  |  |
| sh-SATB2-AS1#1 | 5’-GCCACGAGTATATCAGCAACTTTCAAGAGAAGTTGCTGATATAACTCGTGGCTT-3’ | |
| sh-SATB2-AS1#2 | 5’-GGTCAAACCTTCACCCTATTATTCAAGAGATAATAGGGTGAAGGTTTGACCTT-3’ | |
