# Supplementary material for: LncRNA SATB2-AS1 inhibits tumor metastasis and affects the tumor immune cell microenvironment in colorectal cancer by regulating SATB2
Source: Mol Cancer. 2019 Sep 6;18:135. doi: 10.1186/s12943-019-1063-6 (PMC6729021; doi:10.1186/s12943-019-1063-6)
Supplement: Supplementary file 1 — Table S1. SiRNAs and sh-RNAs sequence. Table S2. The list of primers and probes. Table S3. Information of antibodies. (ZIP 44 kb) [file 12943_2019_1063_MOESM1_ESM.zip › Table S2.docx]

**Table S2： The list of primers and probes.**

| **qPCR primers** | | |
| --- | --- | --- |
|  | **Forward Primer** | **Reverse Primer** |
| SATB2-AS1 | CGAATCCCTTCCTCCTCTCC | TCGTCTTAGCCCTTTCCGTT |
| SATB2 | CTTTGCAAGAGTGGCATTCA | GTTGTCGGTGTCGAGGTTTT |
| CXCL9 | CCAGTAGTGAGAAAGGGTCGC | AGGGCTTGGGGCAAATTGTT |
| CXCL10 | GTGGCATTCAAGGAGTACCTC | TGATGGCCTTCGATTCTGGATT |
| U6 | CTCGCTTCGGCAGCACA | AACGCTTCACGAATTTGCGT |
| WDR5 | TGCTGCAACTTCAATCCCCA | GTGTCCCAGATGCGACAGAG |
| GADD45A | TCAGCGCACGATCACTGTC | CCATTGATCCATGTAGCGAC |
| GAPDH | GGGAGCCAAAAGGGTCATCA | TGATGGCATGGACTGTGGTC |
| NM_001172509 | GTCTCTCTCTGCGGCTTGTC | GCCTTTTCACACCTTCGGGA |
| NM_001172517 | AATCAGCCCCAGGGTTGGAC | AAAACGCACAGGGACCTTGA |
| NM_015265 | CTTCTTCCCTGCCCGGTATC | AAAACGCACAGGGACCTTGA |
| **RACE primers** | | |
| **5’ RACE:** | | |
| Long primer | CTAATACGACTCACTATAGGGCAAGCAGTGGTATCAACGCAGAGT | |
| Short primer | CTAATACGACTCACTATAGGGC | |
| Specific primer | GCTGATATACTCGTGGCAAGGGAG | |
| **3’ RACE:** | | |
| Long primer | CGAAAGCGACAAGGCCGTGATCCCGAAAGCTTTTTTTTTTTTTTTTTTTTTTTTTTVN | |
| Short primer | CGAAAGCGACAAGGCCGTGATCCCGAAAGC | |
| Specific primer 1 | TGATTTCGAAGGCCCCAATTAACTATC | |
| Specific primer 2 | TCGGCGAGTGCAAAGTAGCTGC | |
| **primers for ChIP** | | |
|  | **Forward Primer** | **Reverse Primer** |
| C1 | AGACAACTAAGCAAATGGCATACA | AGCAAAGTAGCCTTATCAACTAGA |
| C2 | GAATTCTTAGTGCAAGAGCAGCC | TCTTGCTTCTTGCGGGTGTC |
| C3 | GTTGCGTGGTGGATGCAGAG | CTCAGGGCACAGCTCTGTTGG |
| C4 | TGGATGTTTGTAGAGTTGTATTTCAT | CTGCTGGAAATAATCGCTTCAGT |
| C5 | GGATCTCAGGCCCAAGGTGG | CACATCCTTAACCCGTGGACTG |
| C6 | GACTGTCCCGTTGGATATGACAG | TAAATTACGGCATGGTTCCCAGG |
| C7 | CTACATCAGCCTCACTGGAACTG | AAAGCCGAATCCGCAGAAGTTAT |
| C8 | AATTACCTGCTCAAAGAGCCACC | AATTACCTGCTCAAAGAGCCACC |
| C9 | TGCGGTGGGCACTTGGTTTAC | GGGTGGCACAACCTCTCCCAG |
| C10 | GGGTGGCACAACCTCTCCCAG | GGGTCCTGTGTTTGAAAGCAAC |
| **primers for BSP** | | |
|  | **Forward Primer** | **Reverse Primer** |
| R1 | GTTTTTTTTTTGGGATTTTTT | ATTACACCTCATATCCCTCC |
| R3 | GGGGAGAGAGATAAGTATAGAGGGTT | TCTAACTTCCCAAACCAAAAT |
| R4 | GTAAGAAAGTTTGGGTAGTTGGA | ATCCCTCTAACCCAAACAAA |
| **FISH probes** | | |
| SATB2-AS1 | TGCAACAACGGGGAATTAGGAG | |
| **ISH probes** | | |
| SATB2-AS1 | 5’-DIG-GTGGGTAAGTCGGGAGCAGAATAATGAAGC-DIG-3’ | |
| **ChIRP probes** | | |
|  | **Sense (5’-3’)** | |
| Probe 1 | TCTTTGCCTGATGACTCAAC-/3bio/ | |
| Probe 2 | GTGTGAACATCGACTAAGCC-/3bio/ | |
| Probe 3 | CTGATATACTCGTGGCAAGG-/3bio/ | |
| Probe 4 | AATCAAGGACTAAGGTGAGC-/3bio/ | |
| Probe 5 | CACACAATCTTGATTCCCAG-/3bio/ | |
| Probe 6 | GAAAGGCCGATGCTCAGAGC-/3bio/ | |
| Probe 7 | TAGGGTGAAGGTTTGACCCG-/3bio/ | |
| Probe 8 | GTAAGTCGGGAGCAGAATAA-/3bio/ | |
| Probe 9 | TGTTAGCGCAATGCGCACTC-/3bio/ | |
| Probe 10 | ACAGATAGTTAATTGGGGCC-/3bio/ | |
| Probe 11 | CGAAACGCCGGTTCTGTAAA-/3bio/ | |
| Probe 12 | AATTAGGAGGGAGCTTGGCC-/3bio/ | |
