# Supplementary material for: LncRNA SATB2-AS1 inhibits tumor metastasis and affects the tumor immune cell microenvironment in colorectal cancer by regulating SATB2
Source: Mol Cancer. 2019 Sep 6;18:135. doi: 10.1186/s12943-019-1063-6 (PMC6729021; doi:10.1186/s12943-019-1063-6)
Supplement: Supplementary file 1 — Table S1. SiRNAs and sh-RNAs sequence. Table S2. The list of primers and probes. Table S3. Information of antibodies. (ZIP 44 kb) [file 12943_2019_1063_MOESM1_ESM.zip › Table S3.docx]

|  |  | **Western blot** | **ChIP** | **RIP** | **IHC** | **IF** |
| --- | --- | --- | --- | --- | --- | --- |
| SATB2 (E8R8H) Rabbit mAb | cell signaling technology #39229 | 1:1000 |  |  | 1:600 |  |
| WDR5 (D9E1I) Rabbit mAb | cell signaling technology #13105 | 1:1000 |  | 1:50 |  |  |
| GADD45 alpha (D17E8) Rabbit mAb | cell signaling technology #4632 | 1:1000 |  |  |  |  |
| Tri-Methyl-Histone H3 (Lys4) (C42D8) Rabbit mAb | cell signaling technology #9751 |  | 1:50 |  |  |  |
| GAPDH Rabbit Polyclonal | proteintech #10494-1-AP | 1:10000 |  |  |  |  |
| Vimentin Rabbit Polyclonal | proteintech #10366-1-AP | 1:1000 |  |  | 1:2000 | 1:50 |
| E-cadherin Rabbit Polyclonal | proteintech #20874-1-AP | 1:1000 |  |  | 1:1000 | 1:25 |
| MMP2 Rabbit Polyclonal | proteintech #10372-2-AP | 1:800 |  |  |  |  |

**Table S3： Information of antibodies.**
