# Supplementary material for: LncRNA SATB2-AS1 inhibits tumor metastasis and affects the tumor immune cell microenvironment in colorectal cancer by regulating SATB2
Source: Mol Cancer. 2019 Sep 6;18:135. doi: 10.1186/s12943-019-1063-6 (PMC6729021; doi:10.1186/s12943-019-1063-6)
Supplement: Supplementary file 2 — Supplementary materials and methods. (DOCX 19 kb) [file 12943_2019_1063_MOESM2_ESM.docx]

**Supplemental Materials and Methods**

**RNA isolation and quantitative reverse transcription polymerase chain reaction**

Total RNAs were isolated from tissues and cells by TRIzol (Invitrogen, USA) according to the manufacturer's instructions. QRT-PCR was performed using SYBR Green PCR Kit (Takara, Japan) on an ABI 7500 System. GAPDH was employed as a control for normalization when detected mRNA and lncRNA expression. Each experiment was repeated at least three times. Primers are listed in Additional file 1: Table S2.

**5’ and 3’** **rapid amplification of cDNA ends analysis**

Total RNA was isolated from HCT-116 cells with TRIzol (Invitrogen, USA) according to the protocol. Rapid amplification of cDNA ends assays were performed using the SMART RACE 5’/3’ Kit (Clontech, USA) according to the manufacturer's instructions. Primers are listed Additional file 1: Table S2.

**In vitro transcription and translation assay**

In vitro transcription/translation of SATB2-AS1 was conducted using a TnT Quick Coupled Transcription/Translation Kit (Promega, USA) and detection was performed using a Transcend Non-Radioactive Translation Detection System (Promega, USA) according to the protocol. Luciferase was used as a positive control and no RNA template was used as a negative control. The expression reaction produces significant quantities of biotinylated lysine residues proteins as detected by Transcend Non-Radioactive Translation Detection System. After SDS-PAGE and electro-blotting, the biotinylated proteins can be visualized via binding of streptavidin-horseradish peroxidase (Streptavidin-HRP), followed by chemiluminescence detection.

**Protein extraction and western blot**

Total proteins were extracted from cultured cells using cell lysis buffer. Then, the protein samples were loaded onto 10% sodium dodecyl sulfate polyacrylamide gel electrophoresis. The membranes were blocked with 5 % non-fat milk in Tris-buffered saline and incubated with a specific primary antibody and a secondary antibody. Protein expression was detected by enhanced chemiluminescence kit. Antibodies used in this study were listed in Additional file 1: Table S3.

**Enzyme-linked immunosorbent assay**

CXCL9 levels were determined with MIG/CXCL9 Human ELISA Kit (assay range: 8.23-6000 pg/mL) and CXCL9 levels were determined with IP-10/CXCL10 Human ELISA Kit (assay range: 7.8-500 pg/mL) from Invitrogen (USA), according to the manufacturer’s protocol.

**Immunohistochemistry, RNA in situ hybridization and immunofluorescence**

IHC staining was performed using Dako Envision System (Dako, USA) according to the protocol. The in-situ detection of SATB2-AS1 in tissues was performed using DIG-labeled the SATB2-AS1 probes. The probe sequence was listed in Supplementary Table S1. The IHC-stained tissue sections were scored by two pathologists who were blinded to the clinical parameters, respectively. The percentage of staining intensity (0, negative; 1+, weak; 2+, moderate; and 3+, strong) were recorded. An H-score was calculated using the following formula: [1 × (% cells 1+) + 2 × (% cells 2+) + 3 × (% cells 3+)] × 100. The maximum H-score would be 300, corresponding to 100% of cells with strong intensity. For IF, cells were fixed in 4% paraformaldehyde, permeabilized using 0.5% Triton X-100 and incubated with primary antibody and secondary antibodies according to the manufacturer's instructions. Coverslips were counterstained with DAPI and imaged with a confocal laser scanning microscope (Olympus FV1000). Antibodies information was listed in Additional file 1: Table S3.

**RNA fluorescence in situ hybridization**

FISH assays were performed using Fluorescent In Situ Hybridization Kit (RiboBio, China) according to the protocol. Cy3-labeled SATB2-AS1 probes were designed and synthesized by GenePharma (China). Probes information was listed in Additional file 1: Table S2.

**Subcellular fractionation location**

The separation of the nuclear and cytosolic fractions was using the PARIS Kit (Invitrogen, USA) according to the manufacturer’s instructions.

**Wound healing assay**

HCT-116 and HCT-8 cells (1×10^6^ cells/well) were treated with the indicated reagents, and wounds were made using a 100-μl plastic pipette tip. The size of the wound was measured after 36 h after wound formation, and the wound was imaged. The cell migration area was measured between dashed regions using ImageJ software (Bethesda, USA) and normalized to control cells.

**Transwell migration and matrigel invasion assays**

The migration and invasion assays were conducted using Transwell chambers, which were coated with (invasion assay) or without (migration assay) the matrigel mix (BD, USA) according to the protocol. The homogeneous serum-free single cell suspensions (1 **×** 10^5^ cells**/**well for migration and 5 **×** 10^5^**/**well for invasion, respectively) were added to the upper chambers and medium with 10% fetal bovine serum was added into the lower chambers, then incubated for 24 h. The cells that had migrated or invaded through the membrane to the lower surface were fixed, stained and counted.

**RNA immunoprecipitation assay**

The EZ Magna RNA immunoprecipitation Kit (Millipore, USA) was used according to the manual. Briefly, CRC cells were lysed in RIP lysis buffer. Magnetic beads were pre-incubated with anti-WDR5 or IgG antibody for 30 minutes at room temperature and the cell lysates was immunoprecipitated with beads for 6 hours at 4°C. After that, RNA was purified and detected by qRT-PCR. Antibodies information was listed in Additional file 1: Table S3.

**Bisulfite genomic DNA sequencing**

Genomic DNA was isolated from CRC cells and tissues using the Genomic DNA Purification Kit (Promega, USA), followed by treatment with sodium bisulfite (QIAGEN, USA). Primers for amplifying the SATB2 promoter (R1, R3, R4) bisulfite-modified regions were listed in Additional file 1: Table S2.
