# Supplementary material for: LncRNA SATB2-AS1 inhibits tumor metastasis and affects the tumor immune cell microenvironment in colorectal cancer by regulating SATB2
Source: Mol Cancer. 2019 Sep 6;18:135. doi: 10.1186/s12943-019-1063-6 (PMC6729021; doi:10.1186/s12943-019-1063-6)
Supplement: Supplementary file 3 — Figure S1. Colorectal specifically expressed SATB2-AS1 is associated with prognosis in CRC. Figure S2. SATB2-AS1 regulates metastasis and immune response of CRC. Figure S3. SATB2 is highly expressed in colorectal tissues and correlated with SATB2-AS1 in CRC. Figure S4. SATB2-AS1 regulates metastasis and immune response through SATB2 in CRC. Figure S5. SATB2-AS1 is mainly distributed in the cell nucleus and down-regulation of SATB2 in CRC is partly due to high DNA methylation. Figure S6. SATB2-AS1 binds to the promoter region of SATB2 and recruits WDR5 and GADD45A. (DOCX 4050 kb) [file 12943_2019_1063_MOESM3_ESM.docx]

**Figure S1:**


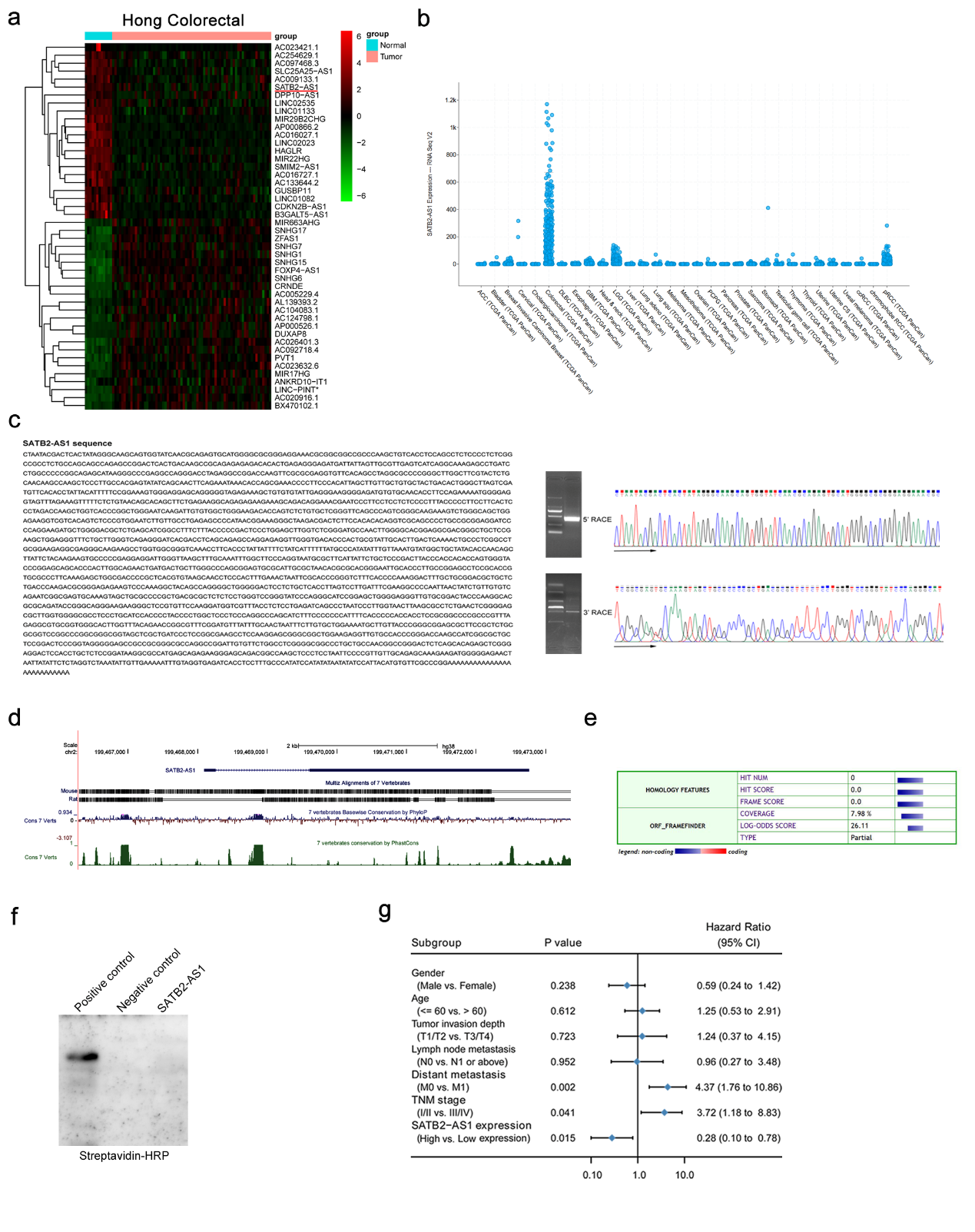


**Figure S1.** Colorectal specifically expressed SATB2-AS1 is associated with prognosis in CRC. **a,** Hierarchical cluster heat map of differentially expressed lncRNAs in CRC and corresponding normal tissues generated from from the GSE9348 dataset in GEO database. Red in the heat map denotes upregulation, green denotes downregulation. The red line indicates SATB2-AS1. **b,** Expression of SATB2-AS1 in different tissues (normal and tumor) and the data obtained from the cBioPortal database. **c,** Representative image of PCR products from the 5’-Race (3’-Race) and the sequence of PCR products are shown. The SATB2-AS1 sequence is shown at the bottom. **d,** Conservation analysis of SATB2-AS1 using the Multiz Alignments, Phylop and PhastCons methods in the UCSC Genome Browser database. **e,** Protein-coding potential analysis of SATB2-AS1 using the Coding Potential Calculator tool. **f,** *In vitro* transcription and translation assay of SATB2-AS1. Luciferase is used as a positive control and no RNA template was used as a negative control. **g,** Multivariable regression analysis was performed in our two CRC cohorts. All the bars correspond to 95% confidence intervals.

**Figure S2:**


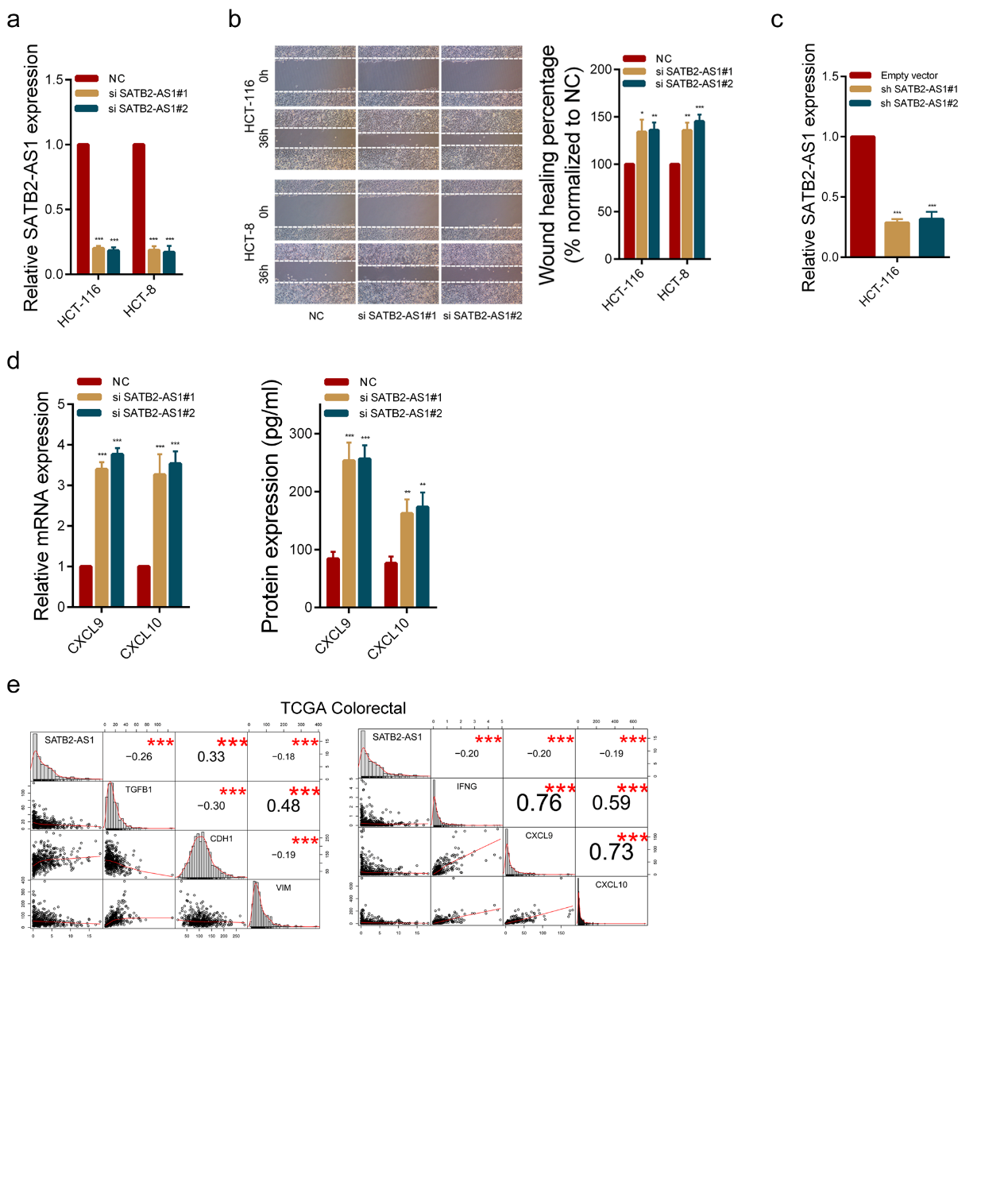


**Figure S2.** SATB2-AS1 regulates metastasis and immune response of CRC. **a,** To verify efficiency of siRNAs, SATB2-AS1 was quantified by qRT-PCR after transfection of SATB2-AS1 siRNAs in CRC cells. **b,** Representative images of wound healing assays performed using CRC cells after SATB2-AS1 silenced. **c,** SATB2-AS1 was quantified by qRT-PCR after transfection of SATB2-AS1 shRNAs in HCT-116 cells. **d,** CXCL9 and CXCL10 were detected by qRT-PCR (left) and ELISA (right) after SATB2-AS1 knockdown in HCT-8 cells. **e,** Scatter plot showing the expression correlation among SATB2-AS1, TGF beta 1, E-cadherin, Vimentin, IFN-γ, CXCL9 and CXCL10 in the TCGA CRC cohort. *, *P* < 0.05; **, *P* < 0.01 and ***, *P* < 0.001.

**Figure S3:**


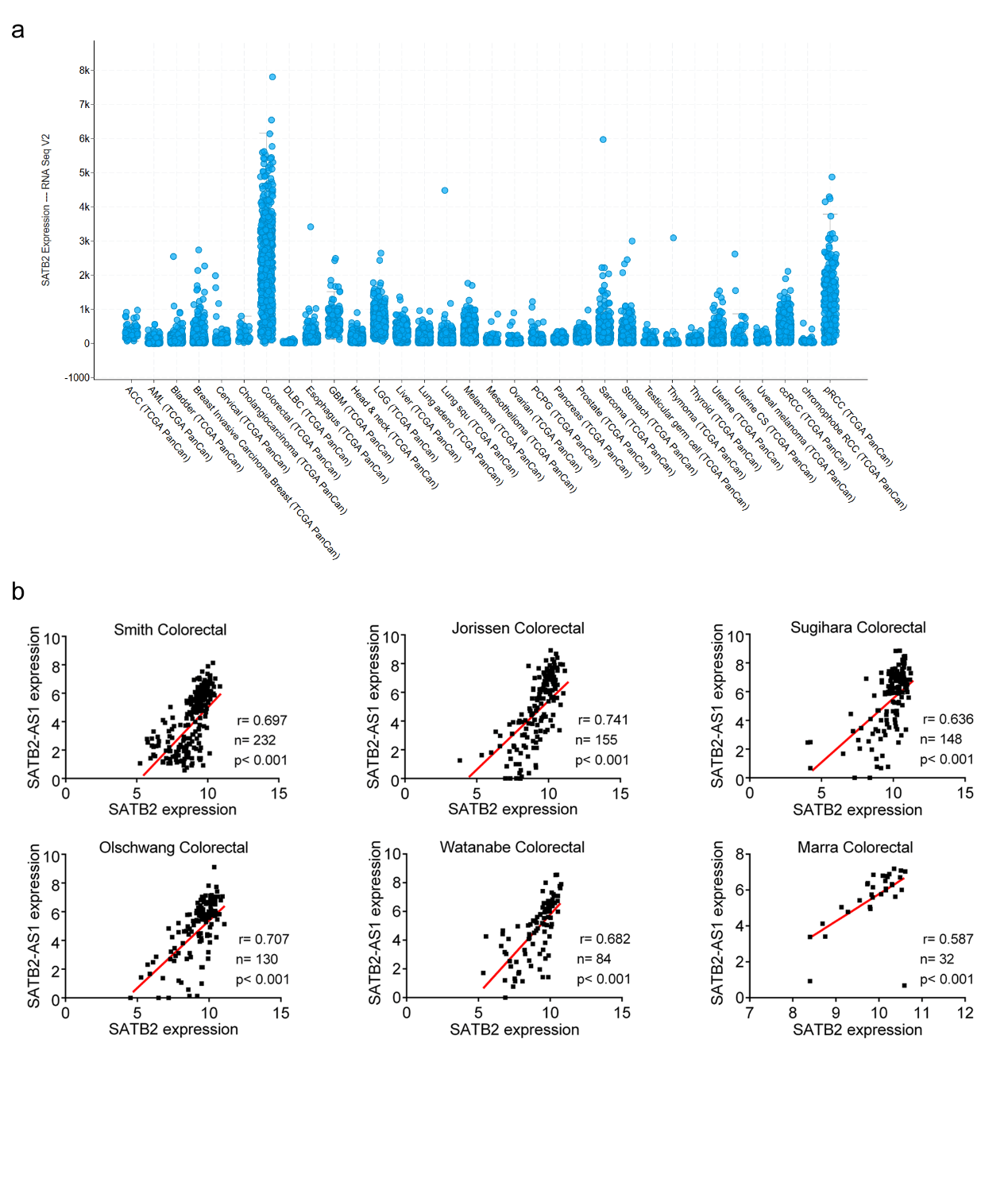


**Figure S3.** SATB2 is highly expressed in colorectal tissues and correlated with SATB2-AS1 in CRC. **a,** SATB2 expression values across a panel of tissues (normal and tumor) and the data obtained from the cBioPortal database. **b,** Correlation analysis of the expression of SATB2-AS1 and SATB2 in indicated CRC cohorts.

**Figure S4:**

**
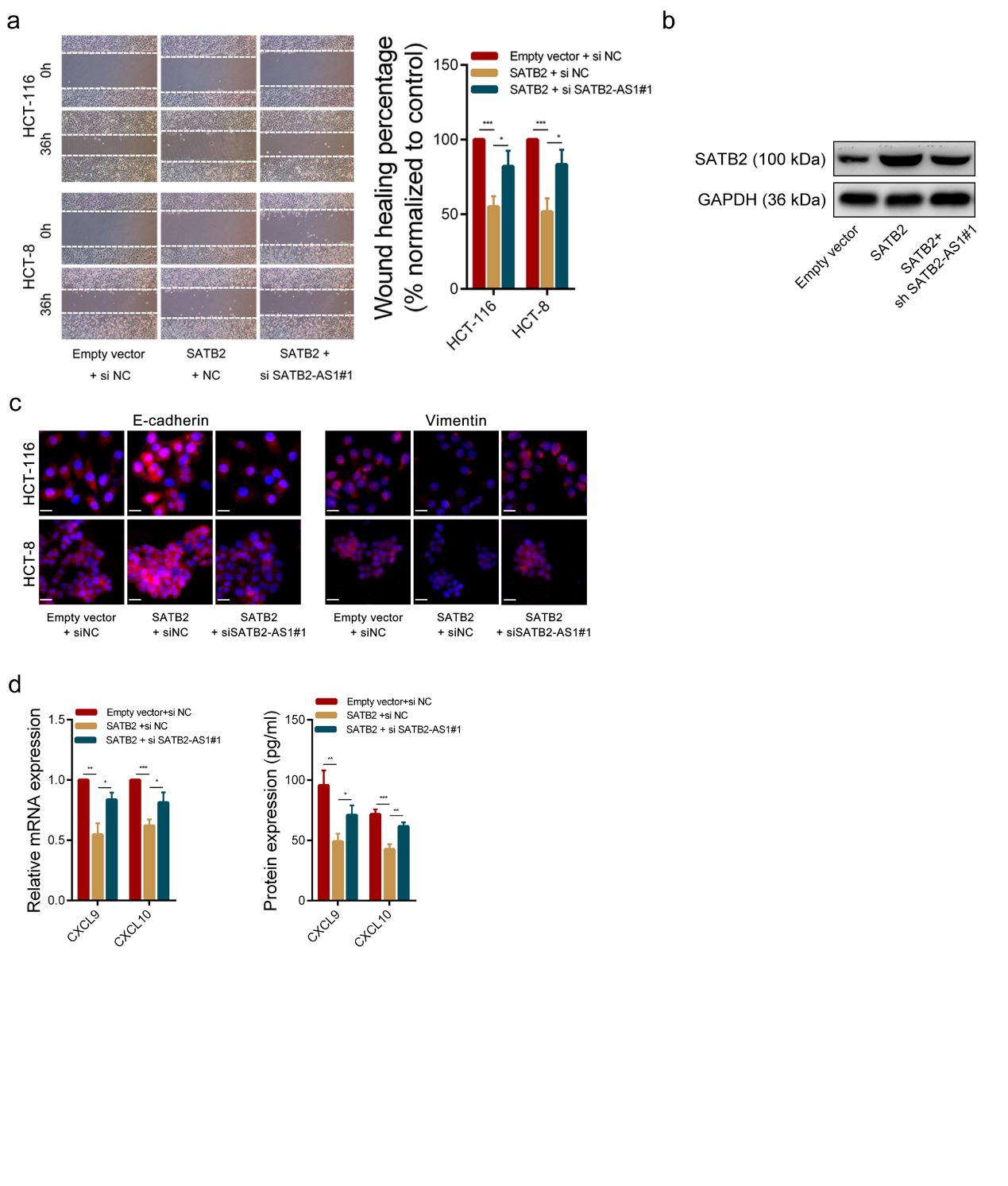
**

**Figure S4.** SATB2-AS1 regulates metastasis and immune response through SATB2 in CRC. **a,** Representative images of wound healing assays performed using CRC cells with indicated treatments. **b,** SATB2 expression was detected by western blot in the corresponding stably transfected cells. **c,** The E-cadherin and Vimentin protein levels were detected by immunofluorescence after indicated treatments. **d,** CXCL9 and CXCL10 were detected by qRT-PCR (left) and ELISA (right) after indicated treatments in HCT-8 cells. Scale bar = 20 μm. *, *P* < 0.05; **, *P* < 0.01 and ***, *P* < 0.001.

**Figure S5:**


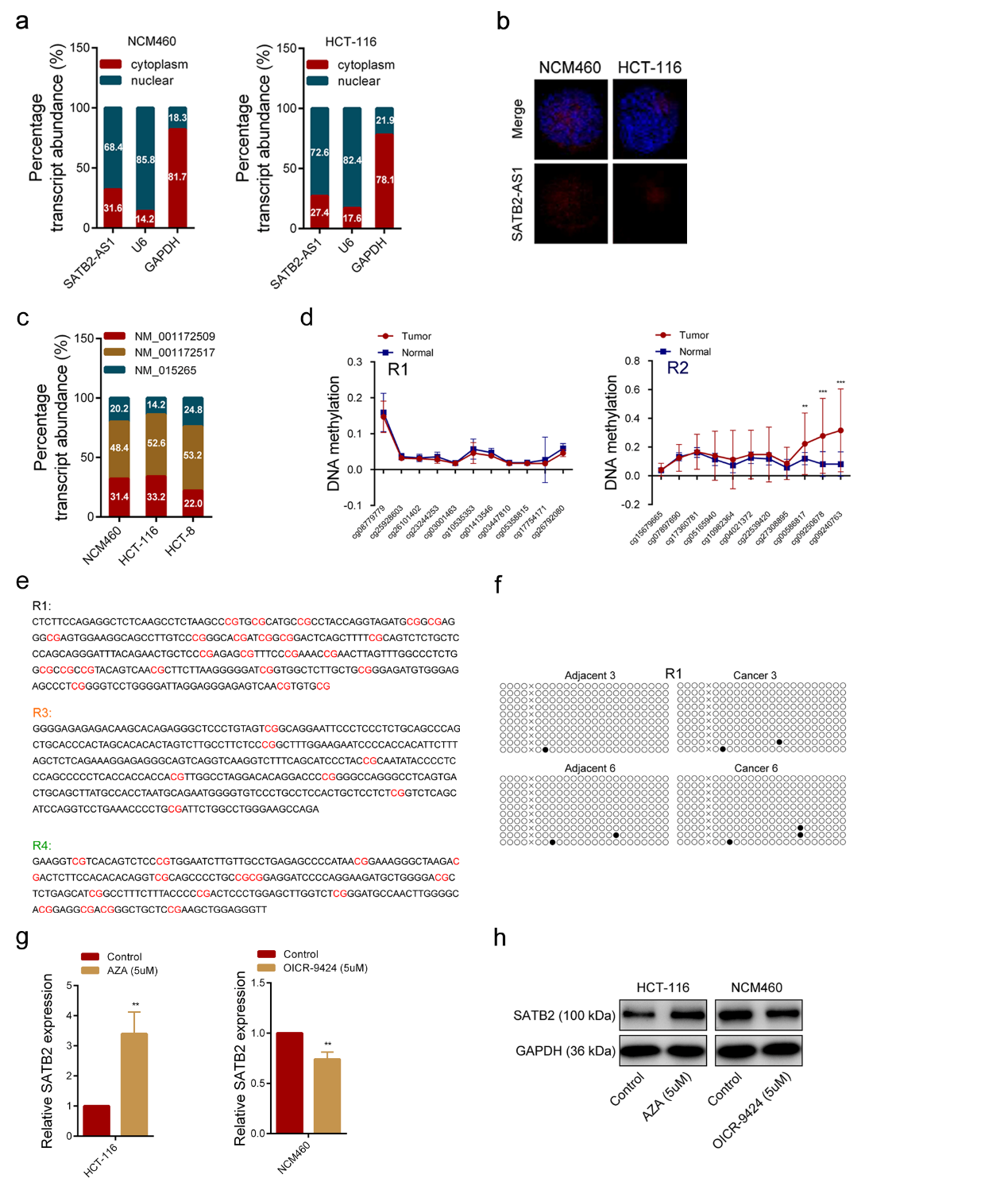


**Figure S5.** SATB2-AS1 is mainly distributed in the cell nucleus and down-regulation of SATB2 in CRC is partly due to high DNA methylation. **a,** Relative SATB2-AS1 expression levels in nuclear and cytosolic fractions of HCT-116 and NCM460 cells. Nuclear controls: U6, cytosolic controls: GAPDH. **b,** Representative FISH images showed the expression of

SATB2-AS1 in HCT-116 and NCM460 cells (red). Nuclei were stained by DAPI (blue). **c,** Relative expression of the three transcripts of SATB2 were evaluated by qRT-PCR in HCT-116, HCT-8 and NCM460 cells. **d,** DNA methylation analysis of SATB2 in CRC and corresponding normal tissues in the TCGA database. Statistical analysis of differences of methylation level between tumors and adjacent normal tissues in R1 and R2 regions. **e,** Sequences of R1, R3 and R4 regions for bisulfite genomic DNA sequencing. CG sites were displayed with red letters. **f,** Bisulfite sequencing was performed to evaluate CpG islands methylation statuses in R1 regions in CRC and adjacent normal tissues. Representative images were presented. Black solid and white hollow circles represent methylated and unmethylated CpG sites, respectively. **g,** SATB2 RNA levels were detected after HCT-116 cells treated with the DNA Methyltransferase inhibitor Azacitidine (5 uM, 48h) and NCM460 cells treated with the WDR5 antagonist OICR-9429 (5 uM, 48h). **h,** SATB2 protein levels were detected after HCT-116 cells treated with the DNA Methyltransferase inhibitor Azacitidine (5 uM, 48h) and NCM460 cells treated with the WDR5 antagonist OICR-9429 (5 uM, 48h). **, *P* < 0.01 and ***, *P* < 0.001.

**Figure S6:**

**
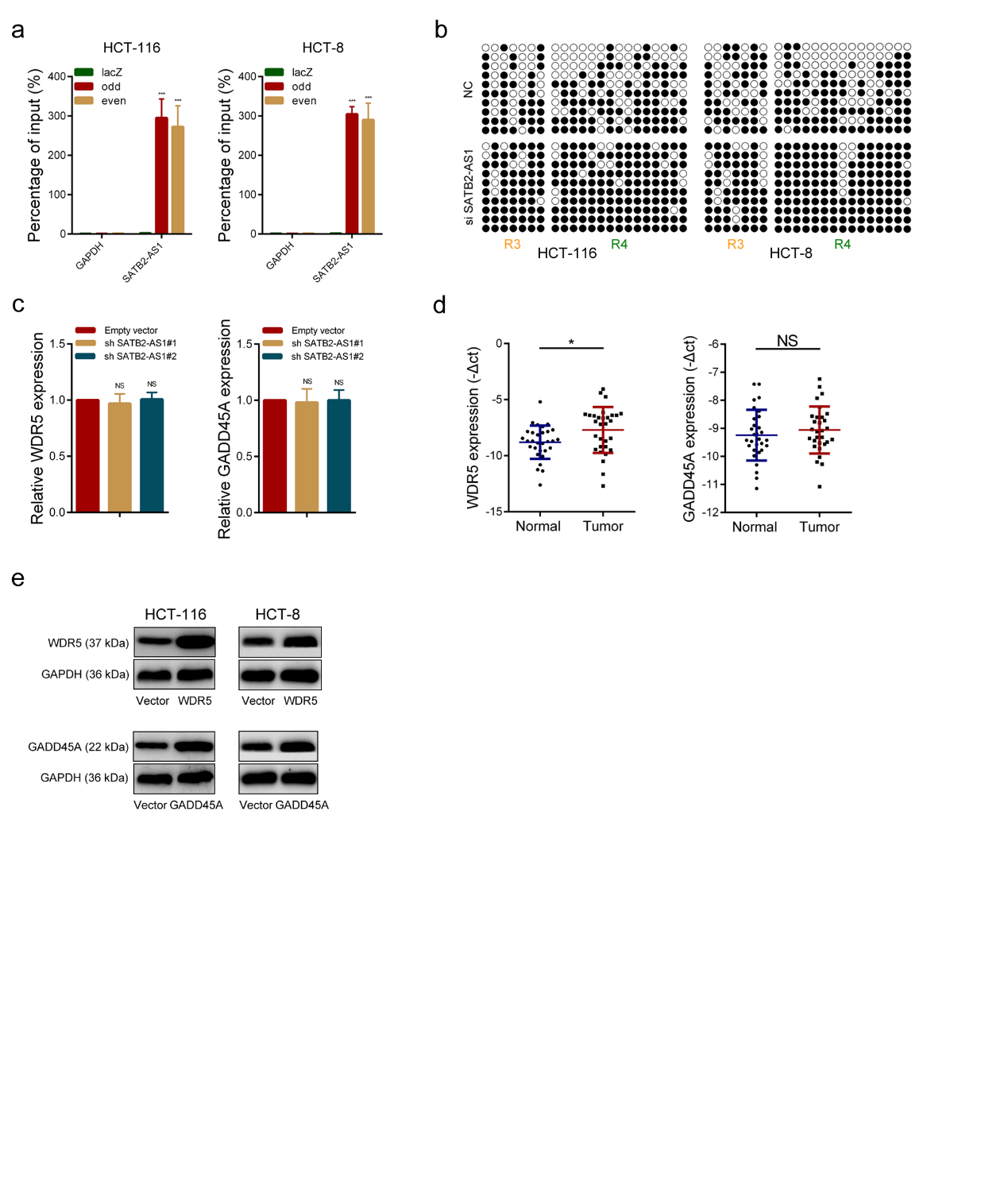
**

**Figure S6.** SATB2-AS1 binds to the promoter region of SATB2 and recruits WDR5 and GADD45A. **a,** SATB2-AS1 targeted probes and negative Laz probes were used for ChIRP assay. Purified RNA was analyzed by qRT-PCR. The results showed that SATB2-AS1 probes specifically pulled down SATB2-AS1. **b,** Bisulfite sequencing was performed to measure the changes of CpG island methylation statuses in R3 and R4 regions in CRC cells after SATB2-AS1 knockdown. Representative images were showed that black solid and white hollow circles represent methylated and unmethylated CpG sites, respectively. **c,** RNA levels of WDR5 and GADD45a in SATB2-AS1 knock down HCT-116 cells and control cells were detected by PCR. **d,** RNA levels of WDR5 and GADD45a in 30 pairs of CRC and adjacent tissues were detected by PCR. **e,** Western blot analyses of WDR5 or GADD45A expression after transfection of WDR5 or GADD45A vectors. *, *P* < 0.05 and ***, *P* < 0.001.
